# Tyrosine Kinase c-MET as Therapeutic Target for Radiosensitization of Head and Neck Squamous Cell Carcinomas

**DOI:** 10.3390/cancers13081865

**Published:** 2021-04-14

**Authors:** Lina Lüttich, María José Besso, Stephan Heiden, Lydia Koi, Michael Baumann, Mechthild Krause, Anna Dubrovska, Annett Linge, Ina Kurth, Claudia Peitzsch

**Affiliations:** 1 OncoRay—National Center for Radiation Research in Oncology, Faculty of Medicine and University Hospital Carl Gustav Carus, Technische Universität Dresden, Helmholtz-Zentrum Dresden—Rossendorf, 01307 Dresden, Germany; lina.luettich@web.de (L.L.); stephan.heiden@dynabind.com (S.H.); Lydia.Koi@uniklinikum-dresden.de (L.K.); michael.baumann@dkfz-heidelberg.de (M.B.); Mechthild.Krause@uniklinikum-dresden.de (M.K.); anna.dubrovska@oncoray.de (A.D.); Annett.Linge@uniklinikum-dresden.de (A.L.); 2 German Cancer Research Center (DKFZ), 69120 Heidelberg, Germany; mariajose.besso@dkfz-heidelberg.de (M.J.B.); ina.kurth@dkfz-heidelberg.de (I.K.); 3 Department of Radiotherapy and Radiation Oncology, Faculty of Medicine and University Hospital Carl Gustav Carus, Technische Universität Dresden, 01307 Dresden, Germany; 4 Helmholtz-Zentrum Dresden-Rossendorf (HZDR), Institute of Radiooncology–OncoRay, 01307 Dresden, Germany; 5 German Cancer Consortium (DKTK) Core Center Heidelberg, 69120 Heidelberg, Germany; 6 National Center for Tumor Diseases (NCT), 01307 Dresden, Germany; 7 German Cancer Consortium (DKTK) Partner Site Dresden, 01307 Dresden, Germany

**Keywords:** radiotherapy, cancer stem cells, c-MET kinase signaling, head and neck squamous cell carcinoma, resistance

## Abstract

**Simple Summary:**

The overall five-year survival rate of patients with loco-regional advanced head and neck squamous cell carcinomas (HNSCC) is only around 40%. The curability of HNSCC with radiochemotherapy was previously found to be associated with clinical and biological parameters including tumor volume, hypoxia, epidermal growth factor receptor expression, and human papillomavirus infection status. Different signaling pathways, e.g., constitutively activated receptor tyrosine kinase signaling, increased DNA damage repair and intracellular defense against reactive oxygen species were identified as factors driving HNSCC progression and its resistance to therapy. c-MET was found to be hyperactivated in HNSCC and has been reported to drive tumor progression, therapy resistance, and metastatic spread. Here, we investigated the therapeutic potential of c-MET targeting strategies for HNSCC radiosensitization and discovered putative resistance mechanisms impeeding success of therapeutic intervention. This study highlights the importance of detailed knowledge about biological mechanisms and regulatory networks for future patient stratification and individualized treatment concepts.

**Abstract:**

The receptor tyrosine kinase c-MET activates intracellular signaling and induces cell proliferation, epithelial-to-mesenchymal-transition and migration. Within the present study, we validated the prognostic value of c-MET in patients with head and neck squamous cell carcinoma (HNSCC) treated with radio(chemo)therapy using the Cancer Genome Atlas database and found an association of increased *MET* gene expression and protein phosphorylation with reduced disease-specific and progression-free survival. To investigate the role of c-MET-dependent radioresistance, c-MET-positive cells were purified from established HNSCC cell lines and a reduced radiosensitivity and enhanced sphere-forming potential, compared to the c-MET-depleted cell population, was found in two out of four analyzed cell lines pointing to regulatory heterogeneity. We showed that c-MET is dynamically regulated after irradiation in vitro and in vivo. Interestingly, no direct impact of c-MET on DNA damage repair was found. The therapeutic potential of eight c-MET targeting agents in combination with irradiation demonstrated variable response rates in six HNSCC cell lines. Amongst them, crizotinib, foretinib, and Pha665752 exhibited the strongest radiosensitizing effect. Kinase activity profiling showed an association of crizotinib resistance with compensatory PI3K/AKT and MAP kinase signaling. Overall, our results indicate that c-MET is conferring radioresistance in HNSCC through modulation of intracellular kinase signaling and stem-like features.

## 1. Introduction

Every year nearly 880,000 malignant neoplasia of the head and neck region are registered worldwide and prognosis depends on tumor stage at diagnosis [1,2]. More than a half of the patients with late stage disease die within five years after diagnosis [3]. Although radiation therapy is a mainstay of treatment for patients with early and locally-advanced HNSCC, around 30% of the patients will relapse with local recurrence or distant metastasis [4,5,6]. Amongst others, therapy resistance and uncontrolled cell growth are driven by constitutively active and oncogenic receptor tyrosine kinases (RTK) like the epidermal growth factor receptor (EGFR) and the hepatocyte growth factor (HGF) receptor c-MET (mesenchymal-epithelial transition factor) in combination with loss of function of the tumor suppressor phosphatase and tensin homolog (PTEN) leading to activation of downstream kinases such as phosphatidylinositol-4,5-bisphosphate 3-kinase (PI3K) [7,8]. These genomic alterations are leading to chromosomal instability, determine tumor heterogeneity and impede therapy response. A major signaling pathway contributing to tumor cell resistance is the PI3K/AKT signal pathway activated through EGFR [9,10]. EGFR and c-MET proteins are closely linked at the cell membrane and activate common downstream signaling pathways such as RAS/RAF/ERK, PI3K/AKT, and JAK/STAT [11,12]. Several clinical studies, including data from our institute, demonstrated that increased *MET* gene expression significantly correlates with reduced locoregional control, decreased overall survival and enhanced distant metastasis after post-operative radio(chemo)therapy, especially in patients with human papillomavirus (HPV)-negative HNSCC, while it does not have prognostic potential in patients treated with primary radio(chemo)therapy [11,13,14,15,16]. HGF binding induces c-MET dimerization and trans-phosphorylation of several tyrosine residues within the C-terminal domain of the β subunit that initiate recruitment of the adaptor protein growth-factor-receptor bound protein 2 (Grb2)-associated binder 1 (Gab1) [17,18] and induces cell motility, growth, and angiogenesis [19,20,21].

Currently available clinical study results provide only little support for the potential of molecular targeted therapies in combination with radio(chemo)therapy to improve survival of patients with locally advanced and metastatic HNSCC. For example, the anti-EGFR antibody cetuximab was the first targeted therapy approved for patients with HNSCC [22]. However, its effectiveness varies between tumor location, stage, and EGFR alteration [23]. For example, results from the recently published De-ESCALaTE HPV trial shows no clinical benefit of cetuximab over cisplatin [24], while in unselected patients with locally advanced HNSCC, cetuximab combined with radiotherapy was inferior to cisplatin-based radiotherapy (ARTSCAN III trial) [25]. Putative resistance mechanisms to EGFR-targeted therapies include mutations in the extracellular domain of *EGFR* and interaction with other RTKs such as c-MET that induces activation of intracellular signaling despite EGFR inhibition [26,27]. c-MET-specific targeted therapies have been tested for non-small cell lung cancer, gastrointestinal cancers, hepatocellular carcinoma, and HNSCC [28,29]. Currently running clinical trials assess the efficacy of selective, oral c-MET inhibitor tivantinib (ARQ 197), capmatinib (INC280), and anti-HGF antibody ficlatuzumab (AV-299) in patients with recurrent, metastatic HNSCC after progression on cetuximab or panitumumab therapy (NCT01696955, NCT02205398, and NCT03422536). Most of the phase II trials failed to show efficacy of c-MET targeting, which might be due to insufficient patient stratification and lack of available predictive biomarkers [30]. This illustrates the urgent need for pre-clinical discoveries investigating the potential of molecular targeting approaches in combination with radiotherapy.

c-MET has been shown to be essential to maintain the subpopulation of cancer stem cells (CSCs) within tumors [31,32]. CSCs are characterized by infinite self-renewal, cellular plasticity, high migratory capacity and therapy resistance. In addition to c-Met, other CSC markers like the adhesion and hyaluronic acid (HA)-binding protein CD44, the amino acid transporter LAT1/CD98, the glycoprotein prominin-1/CD133, and aldehyde dehydrogenase (ALDH) have been identified in HNSCC [13,33,34,35,36,37,38].

Within the present study, we identified altered c-MET RTK signaling in radioresistant and stem-like population of established HNSCC cell lines, in subcutaneous xenograft model in NMRI nu/nu mice and within publicly available patient datasets (TCGA and HIPO-HNC). We found that intracellular c-MET signaling is influencing the clonogenic and sphere-forming potential after irradiation without affecting DNA repair capacity. We could classify HNSCC cells according to their c-MET dependency that was associated with survival after irradiation. We analyzed the efficacy of eight clinically relevant c-MET targeting agents for their radiosensitizing and CSC eliminating potential to assess the hypothesis that c-MET-dependency represents a therapeutically exploitable vulnerability in HNSCC cells. Our results indicate that the sensitivity of HNSCC cell lines to c-MET targeting agents is independent of their c-MET addiction and is more likely to be influenced by other compensatory intracellular kinase signaling pathways such as AKT or ERK1/2.

## 2. Materials and Methods

### 2.1. Cell Culture

Within this study we worked with several established human squamous cell carcinoma (SCC) lines of the head and neck. The FaDu_DD_ [37] cells originate from the hypopharynx; SAS (JCRB Cell Bank, NIBIOHN, Osaka, Japan), Cal33 (DSMZ, Braunschweig, Germany) and UT-SCC-5 cells (DSMZ) from the tongue. The metastatic cell line Detroit562 (CLS, Eppelheim, Germany) derives from pleural effusion of a primary tumor of the pharynx. The keratinocyte cell line HaCaT (DKFZ, Heidelberg, Germany) was used as normal tissue control. These established HNSCC cell lines were cultivated as monolayer with Dulbecco’s modified Eagle medium (DMEM; Biochrom, Cambridge, UK) containing 10% fetal calf serum (FCS, Sigma-Aldrich, St. Louis, MO, USA), 2% HEPES buffer (1 M, Biochrom), 1% non-essential amino acids (NEA 100×, Biochrom), 1% sodium pyruvate (Biochrom), and 1% penicillin/streptomycin (10,000 U/mL, Biochrom). The incubator was adjusted to 37 °C and 5% CO_2_. Cells were passaged usually twice per week after a confluency of 70–80% was reached. Cells were only used for experiments until passage 15 and regularly tested for cell authentication and mycoplasma infection. Irradiated (IR) sublines were generated from indicated parental cell line by selection with at least 15 fractions of 4 Gy and analyzed together with age-matched controls [36].

### 2.2. Colony Formation Assay

For the standard colony formation assay, 1000 cells/well were plated as single cell suspension in 6-well plates and irradiated 24 h after plating with different doses of 2, 4, 6, or 8 Gy of X-rays. After 10 days, colonies were washed with 1× PBS, fixed with 10% formaldehyde and stained with 0.05% crystal violet. Colonies containing more than 50 cells were counted manually using a stereo microscope.

For the formation of three-dimensional colonies 96-well plates were coated with 50 µL of 1% agarose. On top, the cells were embedded in 100 µL Matrigel (1:20, Fisher Scientific, Waltham, MA, USA) covered with 50 µL complete medium. The plates were irradiated 24 h later and after eight days colonies with a diameter of at least 150 µm were counted manually through a microscope or scanned with the Celigo Imaging Cytometer (Nexcelom Bioscience, Lawrence, MA, USA). The plating efficiency (PE) was determined as the ratio between generated colonies and the number of plated cells at 0 Gy (sham control) and is given in %. The survival fraction (SF) describes the relationship between the treatment and control group and its curve is calculated based on the linear-quadratic model with GraphPadPrism software (GraphPad Software, San Diego, CA, USA). Curve comparisons were performed by SPSS software (IBM, Endicott, NY, USA).

For c-MET inhibition in 3D-CFA format 1000 cells per well were plated as described above and treated with the determined inhibitory concentration of 5% (IC5) of Pha665752 (2.52 µM), foretinib (0.627 µM) or crizotinib (0.935 µM). Defined drug concentrations were diluted in media and applicated on top of the cells within the polymerized Matrigel-based matrix 4 h before irradiation.

### 2.3. Cell Irradiation

The cells in culture were irradiated with 200 kV X-rays in single doses of 2, 4, 6, or 8 Gy filtered with 0.5 mm Cu. The Maxishot Y.TU 320 machine (Yxlon International, Comet Group, Flamatt, Switzerland) delivered a dose rate of approximately 1.32 Gy/min at 20 mA. The absorbed dose was measured by usage of the Semiflex ionization chamber (PTW). Daily dosimetry and routine calibration ensured dose homogeneity. The control samples remained sham irradiated.

### 2.4. Viability Assay

The c-MET-targeting compound INCB28060, Pha665752, AMG203, AMG337, foretinib, crizotinib, XL184, and EMD1214063 (Cayman Chemical, Ann Arbor, MI, USA) were experimentally used. To determine the half maximal inhibitory concentration (IC50) of chemical inhibitors 3000–8000 cells of FaDu, SAS, and Cal33 cell line were seeded in 96-well plates and treated with the inhibitors in eight titration steps with a concentration range from 0.1 to 100 µM 24 h after plating. Cell viability was analyzed 24 h, 48 h, and 72 h after treatment either with the colorimetric MTT (3-(4,5-dimethylthiazol-2-yl)-2,5-diphenyltetrazolium bromide) or the luminescent CellTiterGlo assay (Promega, Madison, WI, USA) following the manufacturer’s instructions. The luminescence or colorimetric readout was performed at 560 nm absorbance, with reference at a wavelength of 690 nm using a microplate reader (Tecan, Männedorf, Switzerland).

### 2.5. Western Blot Analysis

2.0 × 10^5^ cells per well were seeded in 6-well plates as monolayers and treated with crizotinib (Cayman) or Pha665752 (Sigma-Aldrich, St. Louis, MO, USA) 24 h after plating. At indicated time points, cells were washed with phosphate-buffered saline (PBS, PAA Laboratories, Pasching, Austria) and lysed with radioimmunoprecipitation assay (RIPA, Santa Cruz Biotechnology). The protein concentration was determined with the bicinchoninic acid (BCA) protein assay kit (Thermo Fisher Scientific, Waltham, MA, USA). Samples with equally adjusted protein concentrations were heated within 4× SDS loading buffer (Bio-Rad Laboratories, Hercules, CA, USA) for 5 min at 95 °C before loading on 4–12% polyacrylamide gels (NuPAGE Bis-Tris Protein Gel, Thermo Fisher Scientific). Protein ladder (10–250 kDa, PageRuler, Thermo Fisher Scientific) was used to determine protein weight (kDa). Separated proteins within the SDS-PAGE gel were transferred to a nitrocellulose membrane using NuPAGE transfer buffer (Thermo Fisher Scientific). After blotting, the membranes were stained with Ponceau S (Sigma Aldrich, St. Louis, MO, USA), washed with PBS-T buffer (1× PBS, 0.1% Triton-X100), blocked with 5% BSA for one hour at room temperature and incubated with the primary antibodies 1:1000 with 5% BSA in PBS-T overnight at 4 °C on a shaking platform. Protein expression was evaluated using following primary monoclonal or polyclonal mouse or rabbit antibodies against c-MET (D1C2 XP, #8198S, Cell Signaling Technology, Danvers, MA, USA), phospho-MET (Tyr1230, Tyr1234, Tyr1235) (Thermo Fisher Scientific, 44-888G), phospho-AKT (Ser473) (D9E, Cell Signaling), AKT (C67E7, Cell Signaling), phospho-p44/42 MAPK (Erk1/2) (Thr202/Tyr204) (D13.14.4E, Cell Signaling, #4370T), phospho-SAPK/JNK (Thr183/Tyr185) (81E11, Cell Signaling, #4668T), SAPK/JNK (Cell Signaling, #9252T), and β-actin (8H10D10, Cell Signaling, #3700T). After washing, the membranes were incubated with horseradish peroxidase (HRP)-linked secondary anti-mouse or anti-rabbit antibody (1:10,000, GE Healthcare, Chicago, IL, USA) for one hour. The chemiluminescent signals were visualized by HRP detection reagent (SuperSignal West Dura Extended Duration Substrate kit, Thermo Fisher Scientific) following the manufacturer’s protocol and acquired through an automated imaging system (ChemiDoc, BioRad or Fusion Fx, Vilber Lourmat, Collégien, France). ImageJ software was used to perform semiquantitative densitometric analysis.

### 2.6. Flow Cytometry Analysis and Fluorescence-Activated Cell Sorting (FACS)

For single cell suspension cells were dissociated with accutase, resuspended in FACS-buffer (PBS, 1 mM EDTA, 1% HEPES and 5% FCS) and incubated for 1 h on ice with the anti-c-MET-FITC antibody (eBioclone 97, Thermo Fisher Scientific, Waltham, MA, USA) or the corresponding isotope control (Mouse IgG1-FITC, clone: P3.6.2.8.1, Thermo Fisher Scientific) in a 1:20 dilution. To discriminate dead cells propidium iodide (PI, 1:500, 1 µg/mL) was added. The samples were analyzed with the flow cytometer (BD FACSCanto™ II, BD FACS Celesta, Becton, Dickinson and Company, Franklin Lakes, NJ, USA) or sorted with the BD FACS Aria III with a 100 µm nozzle. Then, 5% of all cells with strongest or least fluorescence intensity was separated and named as c-MET^+^ or c-MET^−^ populations. The purity of the sorted population was validated based on re-analysis and was determined as >98%. Data were analyzed using FlowJo software (version 7.6.2, LLC, Ashland, OR, USA) and gates were set according to the individual isotype controls.

The co-staining of different surface proteins has been performed in the same way as described above for c-MET. The following direct labelled antibodies were used: CD44-PE (1:100, Miltenyi Biotec, Bergisch Gladbach, Germany), CD98-PE (1:100, Thermo Fisher Scientific), CD133/2-PE (1:20, Miltenyi) and EGFR-PE (1:100). 4′,6-diamidino-2-phenylindole (DAPI, 1 µg/mL) was used to exclude dead cells within the co-staining protocol. Aldehyde dehydrogenase activity was analyzed using Aldefluor assay (Stem Cell Technology, Vancouver, BC, Canada) according to the manufacturer’s protocol.

### 2.7. Immunofluorescence Microscopy

Cells were plated in 8-well culture slides (Merck Millipore, Burlington, NC, USA) at a density of 15,000 cells/well in complete medium and irradiation with 4 Gy for yH2AX assay. After 30 min and 24 h cells were fixed for 10 min in 4% formaldehyde at room temperature (RT), washed in PBS, followed by permeabilization, and blocking with 5% BSA and 0.1% Triton-X100 in PBS for 1 h at RT. The cells were then incubated with primary antibody at 4 °C overnight. Within this study we used anti-phospho-H2AX (Ser139) antibody (dilution 1:250, clone JBW301; Merck Millipore, Burlington, NC, USA) and anti-MET (1:200, Cell Signaling) antibody. After washing, cells were stained with secondary antibody goat anti-mouse IgG (H + L)-AlexaFluor488 (1:400, Thermo Fisher Scientific) and 4′,6-diamidino-2-phenylindole dihydrochloride (DAPI) (1 µg/mL, Sigma-Aldrich) for 1 h. After washing the slides were embedded in mounting medium Mowiol 4-88 (Carl Roth, Karlsruhe, Germany). Fluorescence images were taken with Imager M1 (40× magnification, Carl Zeiss AG, Oberkochen, Germany) with the same exposure time setup in Zen software. Automatic quantification of c-MET expression and yH2AX foci count was performed using ImageJ software. The cell nuclei area from DAPI images were selected and overlayed with the c-MET channel to calculate the mean area in relation to the number of imaged nuclei.

For immunofluorescence detection of c-MET in xenograft tumor sections, 7 µm thick sections were cut with a cryo-microtome (CM1950, Leica Camera, Wetzlar, Germany). Tissue sections were hydrated, blocked with serum-free protein block (Dako Products, Agilent Technologies, Santa Clara, USA) and incubated overnight with anti c-MET primary antibody (1:1000 dilution, ab216574, Abcam, Cambridge, UK) at 4 °C. This was followed by 1 h incubation with AlexaFluor488-labelled secondary anti-rabbit antibody (1:400, Invitrogen, Carlsbad, CA, USA). Cell nuclei were stained with DAPI and sections were mounted with Mowiol. Slides were scanned using the AxioScan slide scanner (Carl Zeiss AG, Oberkochen, Germany)

### 2.8. siRNA-Mediated Gene Knock-Down

The gene knockdown of *MET* was performed by using the following small interfering RNA (siRNA): siMET#1 (5′-GGAGGUGUUUGGAAAGAUA-3′), siMET#2 (5′-GGACCGGUUCAUCAACUUC-3′) and an unspecific siRNA control (scrambled, 5′-GCAGCUAUAUGAAUGUUGUC-3′, Eurofins Genomics, Ebersberg, Germany). 2.0 × 10^5^ cells were seeded per well in 6-well-plates 48 h before transfection. The siRNA stock solution (100 µM) was diluted with 5× siMAX dilution buffer (Eurofins Genomics) to gain a working concentration of 10 µM. Then, 30–50 pM of siRNA was mixed together with Lipofectamine RNAi MAX transfection reagent (Thermo Fisher Scientific) in Opti-MEM serum-reduced media (Thermo Fisher Scientific) according to manufacture protocol. Twenty-four hours after transfection cells were harvested for clonogenic survival analysis and knock-down validation via Western Blot analysis.

### 2.9. Sphere-Formation Assay

Single cell suspension was resuspended in growth factor-defined, serum-free mammary epithelial basal medium (MEBM, PAA Laboratories) containing B27 supplement (Invitrogen), 20 ng/mL epidermal growth factor (EGF, Sigma), 20 ng/mL fibroblast growth factor (FGF, Invitrogen), 4 μg/mL insulin (Invitrogen), and 2 mM L-glutamine (PAA Laboratories). One thousand cells per well were seeded in 24-well-plates with ultra-low attachment surface (Corning Inc., New York, NY, USA). Plates were irradiated with 6 Gy 24 h after plating. Fresh MEBM media containing growth factors was added once a week. After 14 days plates were scanned with Celigo Imaging Cytometer (Nexcelom Bioscience, Lawrence, MA, USA) and formed spheres with a diameter of >100 µm were counted. Sphere-forming potential was calculated as formed spheres per plated cells in percentage.

### 2.10. Xenograft Tumor Growth

The animal facility and the experimental procedures (no. TV33/2014) respect the institutional guidelines, the German animal welfare regulations and the European directive (2010/63/EU). The experiments were performed using 7–14-week-old male and female NMRI (nu/nu) mice obtained from the pathogen-free animal breeding facility (OncoRay, Dresden). To immunosuppress the nude mice further, they received whole body irradiation with 4 Gy (200 kV X-rays, 0.5 mm Cu-filter, ~1.3 Gy/min) 2 to 5 days before tumor transplantation. The generation of subcutaneous xenograft tumors from FaDu, Cal33, and SAS cell lines and the determination of their radiosensitivity through tumor control dose 50% (TCD50) analysis were described in detail in previous publications [39,40,41]. Pieces of source tumors were transplanted subcutaneously into the right hind leg of anesthetized mice (120 mg/kg ketamine, 16 mg/kg xylazine) (*n* = 8–15 per cohort). When xenograft tumors reached a diameter of 7 mm, mice were randomly allocated into the different experimental arms and hind legs were locally irradiated. Local irradiations were given with 200 kV X-rays (0.5 mm Cu-filter) at a dose rate of ~1.3 Gy/min; 1.2 Gy per fraction were given in 10 fractions until a total dose of 12 Gy for Cal33 and 3 Gy per fraction (total dose: 30 Gy) for SAS xenografts within 2 weeks. Cisplatin-based chemotherapy (3 mg/kg b.w.) dissolved in sodium chloride (0.9%) was administrated intraperitoneally once per week before irradiation. Tumor growth was determined with manual caliper measurements for length and width blinded twice a week. Tumors were excised 24 h after the last fraction of irradiation for histological examinations. DNA-microsatellite profile and volume doubling time analysis were used to confirm the identity of all transplanted tumors and quality assurance.

### 2.11. Kinome Profiling

Cal33 and SAS cells were treated with crizotinib (IC10) for 4 h and irradiated with 4 Gy. After 24 h, cells with a confluency of 80–90% were lysed on ice with EDTA-free HaltTM lysis buffer containing phosphatase and protease inhibitor cocktail (Thermo Fischer Scientific) in mammalian extraction buffer (M-PER, 1:100, Thermo Fischer Scientific) and harvested from culture plate by scraping according to manufactures protocol. After centrifugation (10 min, >10,000× *g*, 4 °C) protein solution was quantified using Coomassie Plus (Bradford Protein Assay) assay according to manufactures instructions. PamChip^®^ assays (PamGene International BV, ‘s-Hertogenbosch, The Netherlands) to determine protein tyrosine kinase (PTK) and protein tyrosine kinase (STK) activity were performed at the Genomics and Proteomics Core Facility (Microarray-Unit, German Cancer Center, DKFZ, Heidelberg). The 13 amino acid peptide sequences on the PamChip are derived from substrates that match 80–100% homology to UniPROT IDs. Differentially detected substrates analyzed with the pathway analysis tools from Gene Set Enrichment Analysis (GSEA) or g:Profiler to identify regulatory kinase pathways.

### 2.12. In Silico Gene Expression and Proteome Analysis

Publicly available RNAseq, transcriptome, proteome and clinicopathological parameters from patients with HNSCC were retrieved for The Cancer Genome Atlas (TCGA) cohort [7] through UCSC Xena (https://xenabrowser.net/, accessed on 22 December 2020) and for the HIPO-HNC cohort [42,43] from the Gene Expression Omnibus database (accession GSE117973). Raw expression data were normalized by quantile normalization using R software (version 4.0.3, R Core Team). *MET* transcript level were downloaded with RSEM (RNA-Seq by Expectation-Maximization) software package as log2(x + 1) transformed and normalized counts for further analysis. Replicate-based normalized c-MET phosphorylation at tyrosine 1235 determined by reverse phase protein array (RPPA) was retrieved from the above-mentioned TCGA proteome repository. The prognostic significance of *MET* gene expression and c-MET phosphorylation was determined by the Kaplan–Meier method followed by the Log-rank test using the “Survival” R package. The cut-off values were calculated using the “max-stat” R package with progression-free survival as an end-point for both cohorts.

Comparative gene expression and membrane proteome analysis relating the HNSCC cell lines FaDu and Cal33 after multiple fractions of irradiation (IR subline) with parental as well as ALDH positive with negative population were already published previously and re-analyzed with focus on regulatory c-MET network within this study [33,36].

### 2.13. Statistics

All cellular and molecular biological assays were performed using at least three independent biological replicates, including three technical repeats each. Results are represented as mean value including individual replicates and standard deviation (SD) or standard error of the mean (SEM) as indicated using GraphPad Prim software (version 8). Statistical analysis was performed using students t-test or multivariant two-way ANOVA analysis by Excel or SPSS to calculate the p value. Significant results are indicated with * *p* < 0.05, ** *p* < 0.01, *** *p* < 0.001 or **** *p* < 0.0001.

## 3. Results

### 3.1. High c-MET Expression Characterizes a Radioresistant Subpopulation in HNSCC

**Figure 1:**
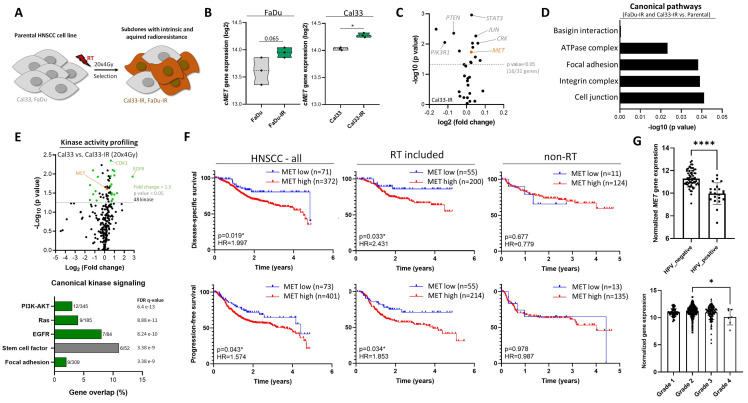
Identification of increased c-MET signaling in radioresistant head and neck squamous cell carcinoma (HNSCC) cell lines and patient data sets. (**A**) Schematic illustration of the generation of multiple irradiated (IR) sublines from established HNSCC cell lines. (**B**) *MET* gene expression analysis in parental compared to IR-sublines from FaDu and Cal33 based on Agilent array data (*n* = 3). (**C**) Comparative gene expression analysis of the MET regulatory gene set (31 genes, BioCarta, Human_RefSeq, ID: M19358) in Cal33- and FaDu-IR cells compared to parental control line demonstrated that 16 out of 31 genes are significantly altered (*n* = 3, *p* < 0.05). (**D**) The intersection between FaDu and Cal33 cell line for differential regulated genes (*p* < 0.05, fold change > 1.5, *n* = 39) in IR-sublines compared to parental control was analyzed for involvement in canonical pathways based on GSEA and g: Profiler webtool. (**E**) Kinase activity profiling (PamGene) identified altered intracellular serine/threonine kinases (STK) and protein tyrosine kinase (PTK) signaling in Cal33-IR cells compared to parental control (*n* = 3). Significantly increased kinase substrates involve, e.g., epidermal growth factor receptor (EGFR), cyclin-dependent kinase 1 (CDK1) and mesenchymal-epithelial transition factor/hepatocyte growth factor receptor (MET/HGFR) (fold change > 1.5, *p* value < 0.05). Upstream kinase prediction and pathway analysis including all significant regulated kinase substrates identified intracellular PI3K/AKT, Ras and EGFR signaling as key pathways altered in Cal33-IR clones. (**F**) Kaplan–Meier analyses validated the prognostic potential of *MET* gene expression for patient stratification and significantly predict disease-specific (*p* = 0.019, HR = 1.997, *n* = 443) and progression-free survival (*p* = 0.043, HR = 1.574, *n* = 474) in the TCGA-HNSCC data set. Subgroup analysis of patients with HNSCC treated with (*n* = 255) or without (*n* = 135) radiotherapy (RT) illustrated that patients with high *MET* expression show a significant reduction for disease-specific survival upon radiotherapy (*p* = 0.033, HR = 2.431). In HNSCC patients treated with surgery or chemotherapy alone *MET* expression does not have any prognostic potential (*p* = 0.677). (**G**) In silico analysis of The Cancer Genome Atlas (TCGA) RNASeq data set for patients with HNSCC (*n* = 517) showed significant negative correlation of *MET* expression with human papillomavirus (HPV) status and tumor grade (* *p* < 0.05, **** *p* < 0.0001).

Comparative transcriptome analysis of pre-irradiated FaDu-IR and Cal33-IR sublines that were previously selected for a cell population with reduced sensitivity, higher CSC marker expression and elevated DNA repair [33,36] identified the MET gene as being significantly upregulated in relation to their parental cell line (Figure 1A,B). In addition to MET itself, we analyzed the regulatory c-MET network (31 genes, BioCarta, Human_RefSeq, ID: M19358) and found 16 genes (51.6%) significantly altered in Cal33-IR subline compared to parental Cal33 cell line (*p* < 0.05) (Figure 1C). For example, STAT3, JUN, and CRK/p38 is up and PIK3R1 as well as PTEN is down-regulated. In parallel, comparative proteome analysis identified 11 proteins up and 4 down-regulated in both analyzed IR sublines, FaDu and Cal33 (*n* = 2, *p* < 0.05) (Figure S1B). Pathway and network analysis including these 15 differently regulated proteins validated the gene expression analysis and found an altered signaling for integrins and focal adhesions with c-MET being a central node in the IR sublines in addition to SRC, CD44, and AKT (Figure 1D and Figure S1B).

The identified altered gene and protein expression within the c-MET kinase signaling in IR sublines suggest that this pathway may be a central regulator for the observed phenotypic and functional alteration. Kinase signaling cascades are regulated by consecutive phosphorylation events. Therefore, we analyzed the kinome of the Cal33-IR sublines using a high-content phospho-peptide substrate microarray system (PamGene) and found 48 differentially activated kinases with a fold change of >1.5 in comparison to parental Cal33 cell line as control (*n* = 3, *p* < 0.05) (Figure 1E). Upstream kinase signaling analysis identified the phosphoinositide 3-kinase (PI3K)/AKT, Ras and EGFR pathway overlapping significantly with the identified peptides (Figure 1E). Thereby, we validated an altered kinase signaling in the IR sublines that may drive the observed altered phenotype and function compared to the parental cell lines.

To validate these pre-clinical findings we analyzed in silico publicly available data sets of HNSCC patients from The Cancer Genome Atlas (TCGA) (*n* = 473) [7] and the HIPO-HNC study (Heidelberg Center for Personalized Oncology-head and neck cancer) (*n* = 77) [42,43] and correlated the MET gene expression with clinicopathological characteristics that influence radiotherapy response. To investigate tumor and therapy-specific effects for patient survival we analyzed the endpoints overall, disease-specific, and progression-free survival and separated patients that received radio(chemo)therapy (*n* = 269) from those that did not receive radiotherapy (*n* = 148). This subgroup of patients received a curative treatment modality either with surgery or concurrent chemotherapy. The progression-free survival data from the TCGA cohort were used to define a cut-off value for RNAseq data to discriminate HNSCC patients with high and low MET gene expression. MET expression appears to have a significant prognostic value for HNSCC patients treated with radiotherapy within the TCGA cohort when analyzing disease-specific (*p* = 0.033, HR = 2.431) and progression-free survival (*p* = 0.043, HR = 1.574). This does not apply to patients treated without radiotherapy (*p* = 0.677, HR = 0.779) (Figure 1F). We used the HIPO cohort for validation. However, the significance level could not be reached here due to low patient number (Figure S1E). In addition, we correlated the MET expression with clinicopathological parameters of HNSCC patients and found an association with human papillomavirus (HPV) as determined by p16 testing, histological grade, tumor location, and nodal (N) status (Figure 1G and Figure S1F). A high MET expression was found in HPV-negative, grade 2 tumors that originate from oral cavity while tumors located in oropharynx express lower MET level. Within TCGA cohort, nine patients have oropharynx cancer with one being HPV positive. However, within the HIPO cohort 36 oropharynx cases are included, from which 22 (61%) are HPV positive. This may explain, at least in part, the lower MET transcript levels in oropharynx cases. Additionally, EGFR may influence c-MET expression and function due to its close relationship. However, we did not find an association of MET expression with amplified EGFR (Figure S1F). Additional subgroup analysis was performed in the HPV-negative cases and confirmed a higher *MET* gene expression within this patient cohort. However, higher MET transcript level was still associated with reduced overall and disease-free survival (Figure S1G). In addition to HPV and EGFR, HGF may influence the prognostic potential of c-MET indirectly. Surprisingly, the survival analysis for HGF gene expression in the TCGA cohort demonstrated an opposing prognostic potential compared to its receptor MET. Despite there is a positive correlation between MET transcript levels and HGF signaling, we found an inverse correlation between HGF transcript levels and HGF signaling indicating that other regulatory mechanisms may be involved (Figure S1H). Importantly, the analyzed TCGA cohort included pre-treatment biopsies from the primary tumors of non-stratified HNSCC patients. Therefore, it is not possible to investigate direct effects induced by radiotherapy.

In summary, this data shows a central role of c-MET in pre-irradiated sublines with increased kinase activity not only for c-MET, but also for EGFR signaling. TCGA and HIPO subgroup analysis of patients that received radiotherapy implies the importance of c-MET and its downstream intracellular signaling for HNSCC progression during radiotherapy (Figure S1A).

### 3.2. c-MET-Expressing HNSCC Cells Are Less Sensitive to Irradiation without Affecting DNA Repair

**Figure 2:**
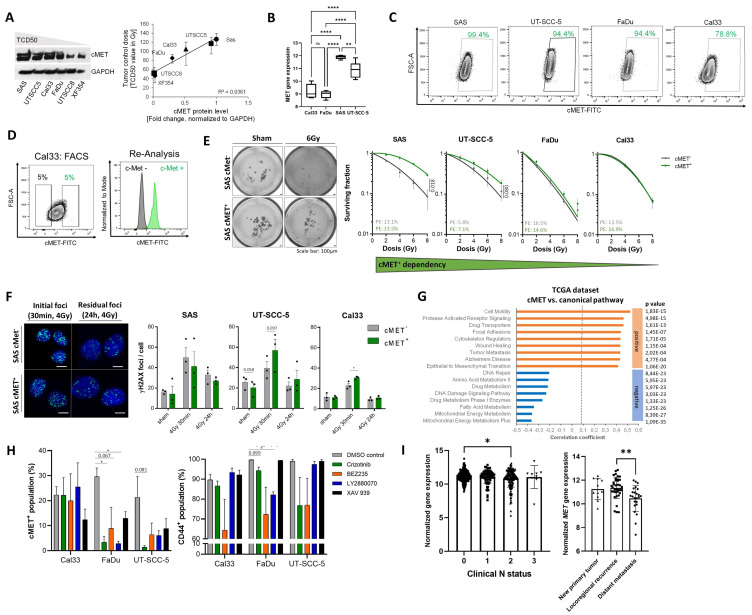
Functional properties of the high c-MET-expressing population in HNSCC. (**A**) Western blot analysis for c-MET protein and correlation to tumor control doses 50% (TCD50) as indicator of tumor radiosensitivity. (**B**) Gene expression analysis of *MET* in Cal33, FaDu, SAS, and UT-SCC-5 cell lines derived from xenograft tumors that were further correlated to previously published tumor control dose 50% (TCD50) values [39,40,41]. (**C**) Proportions of c-MET positivity in different HNSCC cell lines were analyzed by flow cytometry. (**D**) Purification of c-MET high- and low-expressing cell populations (5%) using fluorescence-activated cell sorting (FACS). Reanalysis of the sorted populations demonstrated a purity of >99%. (**E**) The cell-intrinsic radiosensitivity of the purified c-MET-high- and low-expressing population was analyzed with a 3D-matrigel based colony-formation assay in 96-well plates. Dose-response curves illustrate the increased cell survival of c-MET^+^ cells in comparison to c-MET low-expressing cells upon irradiation with different doses (2, 4, 6, and 8 Gy) in SAS and UT-SCC-5 cell lines, while no differences were seen in FaDu and Cal33 (*n* = 3). (**F**) The DNA repair capacity of c-MET-high (c-MET^+^) and -low (c-MET^−^) population upon 4 Gy irradiation was determined through the yH2AX-foci assay (*n* = 3, scale bar = 10 µm). The phosphorylation of histone 2AX indicates DNA double strand breaks and was investigated using immunofluorescence analysis 30 min (initial foci) and 24 h (residual foci) after 4 Gy irradiation (* *p* < 0.05). (**G**) In silico analysis of the TCGA data set correlating the *MET* expression in patients with HNSCC (*n* = 517) with the occurrence of canonical pathways (Molecular Signatures Database (MSigDB), Broad Institute) identified a positive correlation with cell motility, receptor tyrosine kinase (RTK) signaling and focal adhesion, while signaling pathways involved in DNA repair, amino acid metabolism and DNA damage showed significantly negative correlation. (**H**) Chemical inhibition of c-MET with crizotinib (10 µM), mTOR/PI3K with BEZ235, Chk1 with LY2880070, and WNT blockade with XAV 939 (10 nM) for 3 days influenced the percentage of c-MET and CD44-positive population in Cal33, FaDu, and UT-SCC-5. (**I**) *MET* gene expression in primary tumors decreases significantly with increasing nodal status (* *p* < 0.05, TCGA) and is significantly down-regulated in primary tumors of patients with HNSCC with both, increasing nodal (N) status and distant metastasis (* *p* < 0.05, ** *p* < 0.01, **** *p* < 0.0001, ns (not significant)).

Clinical studies from our institution and the German Cancer Consortium (DKTK) demonstrated that dysregulated *MET* expression is associated with locoregional control in HNSCC patients after postoperative, but not after primary radio(chemo)therapy [13,35,44]. We analyzed c-MET protein and gene expression in HNSCC cells highly sensitivity to irradiation, e.g., FaDu and Cal33, in comparison to SAS and UT-SCC-5 with a lower sensitivity and found a significant positive correlation to tumor radiosensitivity that was illustrated by tumor control doses 50% (TCD50) values [45] (Figure 2A,B). However, it is not known if c-MET downstream signaling is directly influencing DNA repair after irradiation. To analyze the impact of c-MET expression on radiosensitivity in HNSCC, we prospectively purified the 5% highest and lowest c-MET expressing cell population in four different HNSCC cell lines using fluorescence-activated cell sorting (FACS) (Figure 2D). Flow cytometry-based analysis demonstrated that 78.8% of Cal33, 94.4% of FaDu, and 99.4% of SAS cells are c-MET positive (Figure 2C). To characterize the intrinsic radiosensitivity of c-MET^+^ and c-MET^−^ cell population, 1000–1500 sorted cells per well were plated under single cell conditions within matrigel in 96-well plates for 3D-colony formation assay and irradiated with increasing doses (2–8 Gy). The fraction of cells surviving irradiation was significantly higher for the c-MET^high^ population in SAS and UT-SCC-5 cell lines in comparison to c-MET^low^ cells. However, we did not observe differences in FaDu and Cal33 cells indicative for a heterogenous c-MET dependency between the tested HNSCC cell lines (Figure 2E). Similar results we obtained in vivo, radioresistant SAS and UT-SCC-5 xenograft tumors exhibit significantly higher *MET* expression compared to more sensitive Cal33 and FaDu tumors while *HGF* is not differential regulated (Figure 2I and Figure S2C).

DNA double strand breaks (DSB) induced by ionizing radiation can be quantified using the yH2AX foci assay. We quantified initial yH2AX foci 30 min after irradiation with 4 Gy, which are indicative for immediate damages, and residual, unrepaired foci 24 h after irradiation. These foci counts were used to calculate DNA repair kinetics from isolated c-MET^+^ and c-MET^−^ cells using immunofluorescence analysis (Figure 2F and Figure S2A,B). However, we did not observe any significant differences between the two purified populations at both time points in the SAS and UT-SCC-5 cell lines. Actually, we found significantly more initial yH2AX foci 30 min after irradiation in c-MET^+^ cells from Cal33 and UT-SCC-5 suggesting a higher irradiation-induced DNA damage. We cannot exclude additional effects involving rapid DNA repair in Cal33 and UT-SCC-5 cells, known to be proficient for homologous recombination (HR) repair, while SAS cells are HR deficient. To investigate how c-MET may determine tumor radiosensitivity in patients with HNSCC, we correlated the *MET* gene expression with occurrence of canonical pathways in silico within the TCGA dataset (*n* = 473). According to our functional analysis, we found that *MET* expression negatively correlates with genes regulating DNA damage repair while it is positively associated to genes involved in cell motility, metastasis, and epithelial-mesenchymal transition (EMT) (Figure 2G). This indicates that c-MET-induced resistance to irradiation is mediated by stem-like features and may explain the association of *MET* expression with the occurrence of distant metastasis in HNSCC patients. To validate the close relationship of c-MET with stemness and intracellular kinase signaling without affecting DNA repair, we treated Cal33, FaDu, and UT-SCC-5 cells with the tankyrase inhibitor XAV 939, the Chk1 inhibitor LY2880070, and the mTOR/PI3K inhibitor BEZ235 for 72 h, and analyzed the percentage of c-MET and CD44 cell populations with flow cytometry. Interestingly, we found that c-MET targeting with Crizotinib and PI3K inhibition with BEZ235 reduced these two populations significantly, while the inhibition of Chk1 and WNT blockade does not (Figure 2H). Despite c-MET-driven metastatic spread in HNSCC is described [30,43,44], we found that *MET* is significantly down-regulated in primary tumors of HNSCC patients with distant nodal metastasis in comparison to nodal negative patients (Figure 2I). The MET pathway can be regulated by different mechanisms, such as the transcription factor ETS1. Transcriptome analysis in HNSCC xenografts and TCGA dataset revealed a significant positive correlation between MET transcript levels and ETS-1 suggesting a possible auto-regulatory loop that may lead to intrinsic MET activation by G-protein-coupled receptors (GPCRs) or other mechanisms independently of HGF such as TGF-β, Src, or MAPK pathways. In addition, we the c-Myb transcription factor network activation that induces *ETS-1* gene involved (Figure 2D). To briefly summarize, the analyzed HNSCC cell lines show a heterogeneous radiosensitivity depending on c-MET abundance without a correlation to DNA repair that was validated within the HNSCC dataset in TCGA.

### 3.3. The c-MET-Expressing Population in HNSCC Is Dynamically Regulated upon Irradiation and Is Characterized by Stem-Like Features

**Figure 3:**
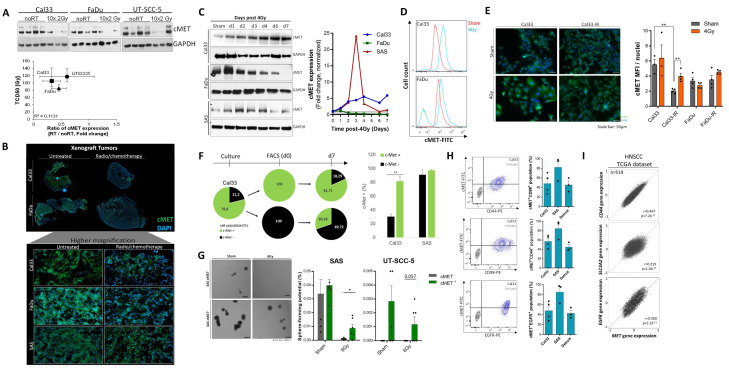
Cellular plasticity and self-renewal properties of the c-MET-population in HNSCC. (**A**) c-MET protein expression within Cal33, FaDu, and UT-SCC-5 xenograft tumors that were treated with 10 fractions of 2 Gy within two weeks. Correlation of tumor control doses 50% with ratio of c-MET protein in irradiated cohort to sham control. (**B**) Immunofluorescence-based evaluation of c-MET expression in s.c. xenograft tumors originating from Cal33, FaDu, and SAS that were treated with cisplatin-based radio/chemotherapy in 10 fractions after randomization when tumors reached a diameter of 7 mm (*n* = 8–15). Tumors were fixed 24 h after last fraction and the formalin-fixed paraffin-embedded (FFPE) tissue was cut into 4 µm sections and stained with anti-c-MET antibody. (**C**) Single dose irradiation with 4 Gy modulates the c-MET protein expression in Cal33, FaDu, and SAS differently in a time-dependent manner within one week. Semi-quantitative analysis of this Western blot using ImageJ demonstrated a dynamic up-regulation of c-MET in Cal33 and SAS as well as a down-regulation in FaDu cells (*n* = 2). (**D**) Flow cytometry-based analysis validated the irradiation-induced upregulation of c-MET on cell membrane 2 days after irradiation with 4 Gy. The depicted overlay histogram compares sham control (red) and post-irradiated cells (blue) for Cal33 and FaDu labeled with c-MET-Alexa488 antibody. (**E**) Immunofluorescence analysis illustrates an increased c-MET expression 5 days upon 4 Gy irradiation and a reduced c-MET signal in IR subclones. c-MET membrane expression and intracellular localization was visualized using an anti-c-MET-specific primary antibody recognized by an AlexaFluor488-labeled secondary antibody (green). The nuclei were stained with 4′,6-diamidino-2-phenylindole (DAPI, blue, scale bar = 50 μm). Quantification of c-Met expression per cell was performed using ImageJ analysis through measurement of mean pixel intensity. (**F**) Cell conversion and plasticity of FACS-purified c-MET-high and low populations in Cal33 and SAS was analyzed 7 days after sorting and seeding of 27,000 cells per well within 6-well-plates by flow cytometry; 100% pure Cal33 c-MET^+^ culture reduces c-MET^+^ proportion to 81.7% after 7 days in culture, while pure c-MET^−^ culture increases c-MET^+^ population to 30.3%. SAS cell line showed to be more plastic than Cal33 and switches back to nearly original proportion of 90% c-MET^+^ cells within 7 days. (**G**) FACS-purified c-MET-high and low population from SAS and UT-SCC-5 cell line were plated under non-adhesive, single cell conditions into low-attachment plates with growth factor defined mammary epithelial basal medium (MEBM). Plates were scanned 14 days after plating with imaging cytometry and formed spheres with a size >100 µm were counted manually. ImageJ was used to calculate sphere-forming potential for each population indicative for self-renewal and stem-like capacity (*n* = 3, * *p* < 0.05). (**H**) Co-expression of c-MET with other cancer stem cells (CSC) and radioresistance markers such as CD44, CD98, and EGFR was analyzed using multicolor flow cytometry (BD FACS Celesta) and illustrates about 50% co-expression in the tested cell lines Cal33, SAS, and Detroit562. (**I**) In silico analysis of TCGA RNASeq data set for patients with HNSCC (*n* = 517) demonstrated significantly, positive correlation of *MET* gene expression with *CD44*, *SLC3A2,* and *EGFR* (Pearson correlation) (* *p* < 0.05, ** *p* < 0.01).

Within our previously published study we found that several CSC markers are dynamically regulated in a dose- and time-dependent manner upon irradiation and that acquisition of a stem-like phenotype may turn HNSCC cells into an irradiation insensitive state [36]. Therefore, we determined the response of c-MET protein expression within FaDu, SAS and Cal33 xenograft tumors that were treated with a cisplatin-based radio/chemotherapy in 10 fractions with 1.8–3.0 Gy per fraction in two weeks. We found that the treatment reduces c-MET protein expression in Cal33 and FaDu xenograft tumors harvested 24 h after the last fraction. However, no differences were seen in SAS-derived xenograft tumors that may explain, at least partially, the known radioresistant phenotype (Figure 3A,B and Figure S3A,B). To further characterize the time dependent-dynamics of c-MET expression after a single dose irradiation with 4 Gy in HNSCC cell lines, Western blot, immunofluorescence, and flow cytometry analyses were performed. Cells were analyzed daily during a week in order to prevent cell passaging effects. As a result, irradiation leads to upregulation of c-MET in all three cell lines with different time kinetics. While irradiation led to a fast upregulation of c-MET in FaDu cells with a maximal signal seen one day after irradiation, the c-MET expression in SAS cells reaches its maxima on day 2 and in Cal33 on day 6 (Figure 3C). This dynamic effect was validated five days after 4 Gy single dose irradiation by flow cytometry (Figure 3D) and immunofluorescence analysis (Figure 3E) in order to discriminate membrane from intracellular expression. In summary, we found that the putative CSC-marker c-MET is differentially regulated between 24 h and one week after irradiation. However, the expression level tends to equilibrate back to baseline level within a certain period of time (Figure 3F). Moreover, we observed dynamic adaptation when c-MET-high and -low expressing cell populations were purified using fluorescent-activated cell sorting (FACS) in culture. Pure c-MET^−^ and c-MET^+^ populations of Cal33 and SAS cell line were cultured for one week and membrane c-MET expression was determined by flow cytometry. Cal33 cells are composed of 21.2% c-MET^−^ and 78.8% c-MET^+^ cells (ratio = 1:3.7). After purification and culture, the proportion of c-MET^−^ (69.7%) to c-MET^+^ (30.3%) within c-MET^−^ cultures equilibrates back to a ratio of 2.3:1. About 30% of c-MET^−^ cells re-gain c-MET expression. The c-MET^+^ culture resembles the proportion of the original culture (ratio = 1:4.5) with 18.3% c-MET^−^ cells and 81.7% c-MET^+^ cells after seven days (Figure 3F). The cell line SAS seems to be more plastic and almost completely recovers to 90% c-MET^high^ phenotype in the c-MET^−^ culture within one week. This dynamic regulation of c-MET within different HNSCC cultures and after irradiation is indicative for adaptive processes involved in stemness and radioresistance.

Functional stem-like properties in vitro can be determined using the sphere-formation assay. Within this analysis cellular properties related to the ability of attachment-independent epithelial cell growth within growth-factor-defined media was assessed. Therefore, purified c-MET^low^ and c-MET^high^ populations of radioresistant SAS and UT-SCC-5 cell lines were plated as single cells under sphere-forming conditions. The determined sphere-forming potential is significantly higher in c-MET^high^ cultures, in particular after 6 Gy irradiation (Figure 3G and Figure S3C). Together with these functional analyses, we investigated the co-expression of c-MET with other known prognostic and resistance markers and performed co-staining experiments with c-MET and CD44, CD98, EGFR, CD133, or Aldefluor. We found that 50–80% of the cells are double-positive for c-MET together with CD44, CD98, and EGFR while only few cells co-express c-MET with CD133 or Aldefluor (Figure 3H and Figure S3E). This finding was validated at gene expression level through analysis of *MET* gene expression in purified ALDH^+^ cells, a putative CSC population, isolated from FaDu cell line. No differences in MET gene expression levels were found between ALDH^+^ and ALDH^−^ population (Figure S3D). Moreover, *MET* expression correlates weakly positive with *ALDH1A3* (*n* = 519, r = 0.206) within the TCGA dataset. Interestingly, *PROM1*, the gene encoding for CD133, negatively correlates with *MET* which is in line with our co-staining data. The other three tested biomarkers *CD44*, *SLC3A2* (encodes for CD98 protein) and *EGFR* show a significantly positive correlation with *MET* in TCGA data, as shown above on protein level in HNSCC cell lines in our flow cytometry experiments (Figure 3I and Figure S3F). All in all, this data validated a role of c-MET in stemness, heterogeneity and plasticity in HNSCC that impacts on radio-responsiveness. Currently, it is not known whether c-MET induction is driving a stem-like phenotype or, vice versa, the acquisition of stem-like features enforces an upregulation of c-MET. However, we hypothesize that the whole CSC population itself is heterogeneous and different CSC populations may compensate functionally within tumors or even transdifferentiate into each other. All in all, this data validated the described role of c-MET for stemness, heterogeneity and plasticity in HNSCC that impacts on radio-responsiveness.

### 3.4. Compensatory Mechanisms Overcoming c-MET-Mediated Radioresponse Uncovered by MET-Specific Gene Knock-Down

**Figure 4:**
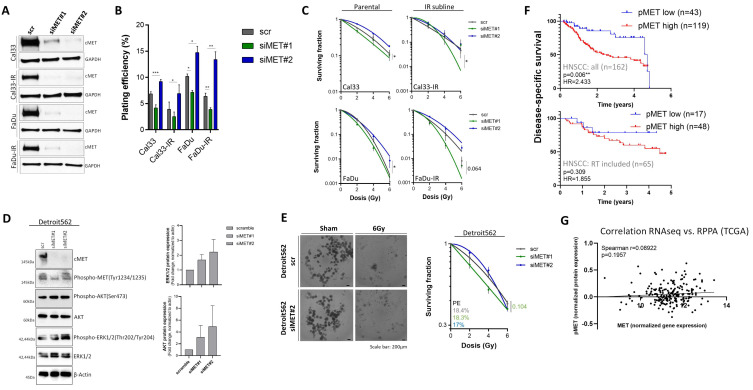
Cellular effects of siRNA-mediated *MET*-specific gene knock-down in HNSCC cell lines. (**A**) Validation of down-regulated c-MET protein expression 24 h upon transfection with 50 pmol of *c-MET* specific siRNA#1 and #2 in comparison to unspecific scrambled control (scr). Western blot analysis demonstrated highly efficient c-MET (175 kDa) down-regulation in Cal33 and FaDu as well as their irradiated sublines (IR). Glyceraldehyde 3-phosphate dehydrogenase (GAPDH, 35 kDa) was used as internal loading control (*n* = 2). (**B,C**) Clonogenic survival and plating efficiency (b) in standard 2D-colony-assay 10 days after c-MET knock-down for Cal33 and FaDu cell line as well as their irradiated sub-lines (IR) in comparison to scramble (scr) control (*n* = 3, * *p* < 0.05, mean ± SEM). Linear-quadratic model of cell survival curves after irradiation with 0, 2, 4, or 6 Gy (c) (*n* = 3, * *p* < 0.05, mean ± SEM). The number of colonies within 10 days is calculated to plated cell number and depicted as plating efficiency. The surviving fraction illustrates the clonogenic survival depending on the irradiation dose and is normalized to plating efficiency. (**D**) Western blot analysis of Detroit562 cells 24 h after *c-MET* knock-down validated the effects on c-MET proteins without affecting c-MET phosphorylation and other downstream kinases such as AKT and ERK1/2. β-actin was used as loading control. (**E**) Clonogenic survival of metastasis cell line Detroit562 which originates from pleural effusion (primary origin: pharynx) 10 days after *MET* knock-down upon transfection with 30 pmol of c-MET specific siRNA#1 or #2 in 3D-matrigel-based cultures treated with increasing doses of irradiation (2, 4, 6, and 8 Gy) (*n* = 3, mean ± SEM). (**F**) In silico analysis of TCGA-HNSCC proteome database (*n* = 162) for the phosphorylated c-MET (Y1235, pMET) protein analyzed via reverse phase protein array illustrates patient stratification with Kaplan–Meier curves. HNSCC patients with high level of pMET have a significantly lower disease-specific survival in comparison to patients with low pMET level in local tumor biopsies (*p* = 0.006, HR = 2.433). Subgroup analysis for HNSCC patients treated with radiotherapy (RT, *n* = 65) does not show significant differences (*p* = 0.309). (**G**) Correlation analysis of MET gene expression with phosphoprotein analysis illustrates no association within the HNSCC-TCGA data set (r = 0.089, Spearman) (* *p* < 0.05, ** *p* < 0.01, *** *p* < 0.001).

The c-MET oncogene activates different intracellular signaling pathways associated with a high clonogenic and tumorigenic potential of tumor cells [46,47,48]. Within our study, we investigated how the IR-subclones with altered intracellular kinase signaling compensates for *MET* knock-down, particularly in combination with irradiation. Therefore, a transient *MET* knock-down in Cal33, Cal33-IR, FaDu, and FaDu-IR cells was performed using RNA interference technology with two different siRNA sequences targeting *MET* mRNA. The knock-down efficiency was evaluated on protein level using Western blot analysis 48 h after transfection and validated a reduction of c-MET protein within knock-down samples <10% compared to the scramble control (Figure 4A). Cells with *MET* knock-down were analyzed for their clonogenic survival after irradiation. We found that the plating efficiency in Cal33 and FaDu cells is already significantly affected after *MET* knock-down indicating an effect on cell proliferation (Figure 4B). siMET#1 decreases the plating efficiency in comparison to scramble control while siMET#2 is showing an opposite effect on clonogenicity. This opposing effect of siMET#1 and #2 was also seen when the knock-down was combined with increasing irradiation doses. However, it is not known why these two different tested siRNA sequences are showing different biological effects. Despite we have efficient c-MET knock-down, we cannot completely exclude off-target effects. However, all four tested cell lines have a significant reduction in c-MET protein and IR-sublines seem to be more dependent on c-MET expression for cell survival after irradiation as illustrated by increased sensitization rate (Figure 4C). We hypothesize that the observed heterogenous cellular response may be indicative of different compensatory, intracellular signaling pathways. To validate this, we performed siRNA-mediated *MET* knock-down in the cell line Detroit562 that originate from metastatic pharyngeal carcinoma. Despite the c-MET protein expression after knock-down was significantly reduced in Detroit562 cells (Figure 4D), we observed only minor cellular effects on clonogenic survival after irradiation (*p* = 0.104, Figure 4E). These results proof the above-mentioned results that purified c-MET^high^ population does not show alteration in DNA repair after irradiation while this cannot explain the differences seen for clonogenic survival (Figure 2D,F).

We hypothesize that the residual c-MET protein within these cells upon knock-down is actively phosphorylated and may induce downstream signaling pathways. Despite we are able to efficiently down-regulate c-MET protein, AKT, and EKR1/2 signaling is still fully active and may be responsible for cell survival after irradiation (Figure 4E). To explore these findings within clinical data, we analyzed the proteomic dataset of patients with HNSCC (*n* = 212) within the TCGA database that contains results for phosphorylated c-MET protein at tyrosine 1235 (Y1235) measured by reverse phase protein array, which is indicative of an activated kinase status. We found that higher level of phospho-c-MET is significantly associated with a decreased progression-free and disease-specific survival in patients with HNSCC (*p* = 0.006, HR = 2.433, *n* = 162). When patients are stratified according to radiotherapy treatment this correlation is not seen (*p* = 0.309, HR = 1.855, *n* = 65) (Figure 4F). These results may be influenced by the tumor stage. 47% of the patients with low c-MET phosphorylation status are in stage III (8/17) while most patients with a high c-MET phosphorylation are diagnosed with stage IV (23/48, 47%). In accordance to these results, we found no correlation between *MET* transcript and phospho-c-MET (Y1235) level. However, we found a significant association between high *MET* transcript levels and lower survival rates. Meaning, the lack of correlation between transcript and phospho-protein might be attributed to a lower number of cases within the proteomics dataset (Figure 4G). Additional studies are needed to investigate putative regulatory mechanisms determining the observed differences in the prognostic potential of *MET* gene expression and phosphorylation status.

### 3.5. Chemical c-MET Targeting for HNSCC Radiosensitization

**Figure 5:**
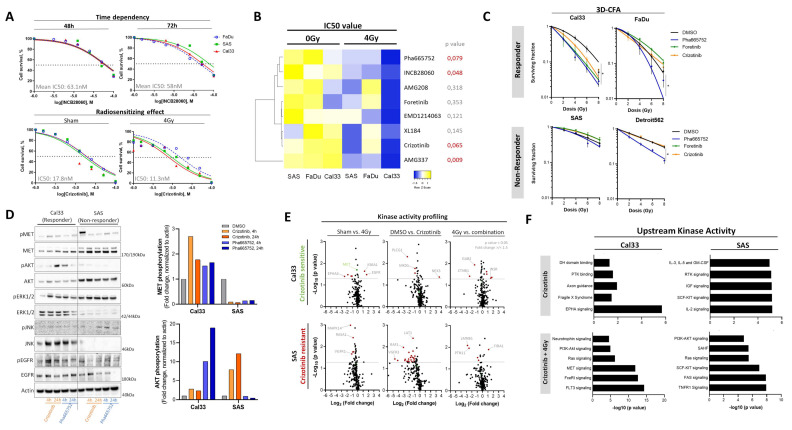
c-MET-specific chemical targeting to induce intracellular sensitization of HNSCC cell lines to ionizing radiation. (**A**) The half maximal inhibitory concentrations (IC50) of eight different c-MET-targeting agents were determined on different time points (24 h, 48 h, and 72 h) with increasing compound concentrations (1–100 µM) and in combination with irradiation (4 Gy) using cell viability assay determining intracellular adenosine triphosphate (ATP) level (CellTiter-Glo, Promega) (*n* = 2). (**B**) Unsupervised cluster analysis illustrates radiosensitizing potential of eight c-MET-targeting chemical compounds in Cal33, FaDu, and SAS cell line. (**C**) Three compounds with significant radiosensitization in cell survival assay were validated within a 3D-colony formation assay including 4 h inhibitory treatment before irradiation with 2, 4, 6, and 8 Gy of X-rays. The number of colonies was examined after 10 days of culture. Depicted are the surviving fractions normalized to plating efficiency of sham control (*n* = 3, mean ± SEM, * *p* < 0.05). (**D**) Activity of different intracellular kinase signaling after 4 h and 24 h of treatment with crizotinib or Pha665752 within the cMET-targeting responsive cell line Cal33 and non-responding line SAS was analyzed by determining specific phosphorylation signals with Western blot (*n* = 2). (**E**) Kinome activity of responding cell line Cal33 and non-responding line SAS was analyzed using capture kinase assay (PTK and STK PamChip) after 4 h treatment with crizotinib (IC10), 24 h after 4 Gy and crizotinib combined with irradiation in comparison to control (sham and DMSO) (*n* = 3). Venn diagrams illustrate identified peptides significantly altered within the treatment group (*p* < 0.05). (**F**) All significantly phosphorylated peptides within a certain treatment group were included to perform pathway analysis with the Molecular Signatures Database (MSigDB) to illustrate altered intracellular upstream protein kinase signaling pathways (* *p* < 0.05).

The above-described data demonstrated that *MET* expression and c-MET cell surface protein abundance is altered in radioresistant and stem-like HNSCC cells. Moreover, we could show that c-MET is dynamically regulated upon irradiation and that a transient knock-down of the *MET* gene is increasing radiosensitivity only within a subset of investigated HNSCC cell lines. We hypothesize that this subset of HNSCC cells with c-MET dependency may be highly sensitive to chemical c-MET-specific targeting. Therefore, we screened eight clinically relevant c-MET inhibitors (foretinib, crizotinib, Pha665752, INCB28060, AMG208, AMG337, EMD1214063, and XL184) in three different HNSCC cell lines (FaDu, SAS, and Cal33) within a dose range of 1–100 µM for different time points (24 h, 48 h, and 72 h) and in combination with 4 Gy irradiation using cell viability assay (CellTiterGlo) (Figure 5A). The determined half-maximal inhibitory concentration (IC50) is indicative for 50% reduction of cell viability. Unsupervised cluster analysis of these IC50 values illustrates the radiosensitizing potential of all eight tested compounds in the three tested cell lines. The highest sensitivity against c-MET targeting was observed for Cal33 and the lowest for FaDu. Three compounds (Pha665752, foretinib, and crizotinib) with significant radiosensitizing effects in the viability assay and already published pre-clinical and clinical data for cancer patients were chosen for further validation with the matrigel-based 3D clonogenic survival assay (Figure 5B). We validated the radiosensitizing potential of all three c-MET targeting compounds in Cal33 and FaDu with Pha665752 showing the highest effects. The cell lines SAS and Detroit562 did not respond to c-Met targeting alone or in combination with irradiation (Figure 5C and Figure S4B). Comparable results were obtained when analyzing IR-subclones after 4 h pre-treatment with Pha665752 in combination with irradiation. While parental Cal33 and FaDu cells were responsive, the IR-subclones with altered kinase signaling did not respond to Pha665752 treatment in combination with irradiation (Figure S4A). These results indicate that compensatory intracellular kinase signaling pathways overcome the chemical inhibition of c-MET signaling as it was already demonstrated above for genetic targeting. In addition, we analyzed clonogenic survival of the spontaneously transformed aneuploid immortal keratinocyte cell line HaCaT after treatment with crizotinib in combination with irradiation. This cell line originates from adult human skin and was used to illustrate putative normal tissue reactivity. We found that crizotinib treatment significantly reduced radiosensitivity of these normal tissue cells and may have the potential to prevent normal tissue toxicity of radiotherapy while sensitizing tumor cells in parallel (Figure S4C).

Receptor tyrosine kinases require adenosine triphosphate (ATP) as source for subsequent phosphorylation of downstream targets. Crizotinib and Pha665752 are potent c-MET inhibitors through competitive binding within the ATP-binding pocket that prevent c-MET-induced downstream signal activation, e.g., of PI3K/AKT, MAPK, and STAT. To investigate the underlying molecular mechanisms determining crizotinib sensitivity, we analyzed the activity of different intracellular kinase cascades in the responder cell line Cal33 in comparison to the non-responder line SAS 4 h and 24 h after crizotinib and Pha665752 treatment using Western blot analysis. In Cal33 cells, we found that c-MET targeting slightly increase MET phosphorylation, followed by an induction of AKT, ERK1/2, and EGFR phosphorylation. These late effects may illustrate the activation of compensatory kinase pathways. Within SAS cells, c-MET was hyperphosphorylated initially and reduced after treatment with crizotinib and Pha665752 affecting downstream AKT and EGFR phosphorylation. Moreover, JNK phosphorylation was induced while ERK1/2 was not affected (Figure 5D). These analyses were used to determine treatment time points for a kinase activity profiling within responding Cal33 in comparison to non-responding line SAS cells. We found that irradiation with single dose of 4 Gy induces MET- and EGFR-specific peptide phosphorylation in Cal33. Twenty-four hours treatment with crizotinib led to massive reduction of kinase activity in SAS cells mainly influencing VGFR3 and RAF1 (Figure 5E). The combination of crizotinib with irradiation showed a significantly reduced phosphorylation of GRB2-associated-binding protein 2 (GAB2) and β-catenin (CTNB1) and increased phosphorylation of insulin receptor (INSR) peptide in Cal33 (Figure 5E). This validates the CSC-targeting capacity of c-MET-specific receptor tyrosine kinase inhibitors (TKI) and identified its action through SCF/KIT pathway (Figure 3E). The non-responder cell line SAS reacted to the combination treatment with reduced lamin-B1 (LMNB1) and tyrosine-protein phosphatase non-receptor type 11 (PTN11) phosphorylation while increasing phosphorylation of FIBA1. Upstream kinase analysis of all significant phosphorylated peptides (*p* < 0.05, fold change > 1.5) identified the neurotrophin, PI3K/AKT, and RAS signaling as key resistance mechanisms (Figure 5F and Figure S4D,E). In summary, these data validated the therapeutic potential of c-MET targeting for HNSCC radiosensitization. However, compensatory mechanisms may prevent clinical efficiency and biomarker-driven patient selections may be necessary to proof radiosensitizing potential in patients with HNSCC.

## 4. Discussion

The pleiotrophic role of the receptor tyrosine kinase (RTK) c-MET in cellular processes and its important role for cancer progression suggest that c-MET may be a promising target for anticancer therapy [49,50,51,52]. Most potent c-MET targeting agents are small kinase inhibitors, biological antagonists and monoclonal antibodies that are currently in early phase clinical trials binding either the ligand HGF or c-MET itself [15,16,53]. RTKs such as c-MET are often overexpressed in locally advanced HNSCCs and are associated with therapy resistance and tumor relapse [13,44,54,55]. A meta-analysis including published clinical data from 1724 patients with HNSCC found a significant association of increased *MET* gene expression with poor overall survival while immunohistochemical staining demonstrated a significant increased association of c-MET protein expression with worse relapse-free survival and the presence of regional lymph node metastasis [32]. Moreover, retrospective clinical studies from our institution and other groups found that *MET* gene expression is associated with unfavorable prognosis of HNSCC patients after radio(chemo)therapy [13,44,54]. Similar findings have been published for anti-EGFR therapies for HNSCC patients e.g., using cetuximab. In a retrospective, single center study, Madoz-Gúrpide et al. found *MET* overexpression in 58%, *MET* amplification in 39%, MET protein phosphorylation in 30%, and HGF overexpression in 58% of the analyzed tumors of patients with HNSCC [56]. These and other clinical findings suggest that c-MET may be a promising target for patients with HNSCC in combination with radiotherapy. Therefore, we investigated within the present study the potential of c-MET targeting strategies for HNSCC radiosensitization and focused on cellular plasticity, stemness and DNA repair as major determinants of radiosensitivity in HNSCC. Several published studies, including studies from our group, demonstrated the prognostic potential of CSC-related biomarkers including CD44, CD133, ALDH1A3, and c-MET for patients with HNSCC treated with radio(chemo)therapy [13,32,36,57]. We, as others before, hypothesize that stem-like characteristics overlap, at least partially, with tumor cell intrinsic radioresistance. Consequently, we propose that a high c-MET expression in HNSCC cells, xenografts and/or patients is associated with reduced radiosensitivity and increased stem cell behavior. The characterization of sublines from established HNSCC cell lines selected through multiple fractions of irradiation over a long period of time identified an altered intracellular kinase signaling involving PI3K/AKT, RAS, and EGFR signaling combined with an up-regulation of the *MET* gene. This indicates that acquired radioresistance goes along with altered c-MET signaling in HNSCC cells. To analyze effects of c-MET on cell-intrinsic radiosensitivity we prospectively purified c-MET^high^ and c-MET^low^ cell populations, and found a significantly higher fraction of clones surviving irradiation within the c-MET^high^ population in comparison to c-MET^low^ cells. However, we did not observe any difference of both populations in FaDu and Cal33 cells, indicating that the association of c-MET and radioresistance differs between the different HNSCC cell lines. Until now, it is not fully understood how c-MET is affecting radiosensitivity. A recently published study by Bensimon et al. applied quantitative phosphoproteomic technique and identified a novel c-MET phospho-site that bears as substrate for the DNA damage response (DDR) machinery including DNA-dependent protein kinase (DNA-PK), nuclear mitotic apparatus protein 1 (NUMA1) and checkpoint kinase 1 (CHEK1) in c-MET addicted cancer cell lines [58]. We analyzed the DNA repair capacity of purified c-MET^high^ and c-MET^low^ cells within the c-MET-dependent cell line SAS and UT-SCC-5 using the yH2AX assay. Surprisingly, we did not find any differences in initial and residual yH2AX foci reflecting DNA double strand breaks after irradiation with 4 Gy. This was validated within the TCGA data set, were we found that *MET* expression is positively associated with stem-like features and negatively with DNA repair mechanisms. To shed further light on this, we investigated the effect of irradiation on c-MET expression. Therefore, we analyzed the dynamic changes in protein expression after treatment of HNSCC cell lines with 4 Gy single dose and found an >20-fold enrichment in SAS cells within 2 days that equilibrates to baseline level within one week. Cal33 cells increase c-MET >6-fold until day 7 while c-MET level in FaDu cells even decline. This heterogeneous, cell line dependent response of c-MET to irradiation was proven with flow cytometry and immunofluorescence analysis and is fitting to the identified c-MET dependency influencing the cell-intrinsic radiosensitivity of SAS and UT-SCC-5 cells. These results are in-line with a previously published study that investigated the stem-like characteristics of c-MET^pos^ cells within primary HNSCC cultures using limiting dilution analysis in immunocompromised NOD/SCID mice [32]. The authors determined the stem-cell frequency within the primary HNSCC culture model SJHN-1 containing one putative CSC within 943 tumor cells as significantly higher for the c-MET^pos^ population compared to 1/27,093 for the c-MET^neg^ population. In addition, the authors determined the stem-cell frequency of other known CSC-markers and found with 1 tumorigenic cell within 497 cells (1/497) a higher stem-cell frequency in the ALDH^high^ population than in the CD44^+^ population containing only 1 in 7364 [32]. Within this study, we determined the self-renewal properties of the c-MET^+^ population using the sphere-formation assay and found that this population is enriched for sphere-forming cells compared to the c-MET^−^ population in particular after irradiation. We cannot exclude that this effect is independent from c-MET, because co-expression analysis demonstrated that c-MET is highly co-expressed and co-regulated with other known CSC markers such as ALDH, CD98, and CD44. Therefore, we applied siRNA-mediated transient gene knock-down to specifically down-regulate *MET* expression that was validated on protein level. Despite we efficiently down-regulated c-MET protein level, there was no radiosensitizing effect within the investigated HNSCC cell lines while the purified c-MET^high^ population was resistant. Downstream signaling analysis demonstrated that even upon c-MET protein reduction the intracellular kinases are still active, which is a consequence of compensatory signaling pathways overcoming loss of intracellular c-MET signal transduction. When analyzing the proteomic TCGA dataset of patients with HNSCC we found that high level of phosphorylated c-MET, i.e., active c-MET is significantly associated with disease-specific survival. We found a discrepancy of *MET* gene expression, protein expression and kinase activity that was confirmed within the TCGA dataset. *MET* gene expression does not correlate with phospho-MET level. The phosphorylation of the intracellular domain of c-MET can activate the PI3K/AKT, RAS/MAPK, and STAT3 signaling pathway. This tight regulation is lost within HNSCC and abnormal, activated or overexpressed *MET* causes tumor progression, invasion and metastasis [59,60,61,62,63]. Therefore, we hypothesized that c-MET-directed targeting may reduce the stem-like potential and sensitize HNSCC cells to ionizing radiation. However, a previously published study demonstrated that the c-MET targeting agent crizotinib does not increase the efficiency of irradiation in HNSCC xenograft tumors that originate from UT-SCC-14 and UT-SCC-15 cells [64]. These cell lines harbor a *MET* over-expression and *EGFR* amplification that may impact on treatment efficiency [65]. In combination with other tyrosine kinase inhibitors (TKI) several c-MET inhibitors such as GSK1363089, INC280, MGCD516 entered clinical trials for patients with HNSCC as single agent or in combination with EGFR-targeting antibody cetuximab (NCT00725764, NCT02205398, and NCT02219711). Pre-clinical and experimental studies applying chemical inhibition of c-MET in vitro and in vivo demonstrated effects on cell proliferation and apoptosis in HNSCC cells [66,67,68,69]. Amongst all developed c-MET inhibitors Pha665752, a potent ATP-competitive agent, was shown to exhibit the highest potential for radiosensitization in nasopharyngeal cancer cell lines. The observed radiosensitizing effect of Pha665752 might be associated with a persistence of DNA damage that suppresses the phosphorylation and downstream activation of AKT, ERK1/2, and STAT3 [70]. These data support the therapeutic potential of c-MET-specific targeting approaches for HNSCC radiosensitization. Described toxicities associated with inhibition of c-MET related signaling are fluid retention, mucositis, hypophosphatemia, neutropenia, cardiac conduction that were observed in approximately 16% of patients [71]. This data encouraged us to screen additional eight c-MET inhibitors in combination with irradiation on viability of different HNSCC cell lines. We validated the high potency of Pha665752, crizotinib as well as foretinib and proved the radiosensitizing potential within clonogenic survival assay including additional HNSCC cell lines. Interestingly, we found that c-MET significantly sensitized the two cell lines, Cal33 and FaDu, to irradiation, while SAS and Detroit562 did not respond. To unravel the compensatory signaling pathways activated within the non-responding cell lines, we profiled the kinome of two cell lines and compared single agent critzotinib treatment to the combination with irradiation. We found the Fas, RAS, and PI3K/AKT signaling to be altered in the non-responding cell line SAS upon crizotinib treatment in combination with 4 Gy while in Cal33 cells the MET signaling pathway is affected. Therefore, we concluded that other compensatory intracellular kinase signaling pathways influence the efficiency of c-MET targeting and may impede the radiosensitizing potential in HNSCC cells. Lacking efficacy based on the activation of alternative RTK signaling, including EGFR, have already been described in resistant HNSCC cells by others before [72]. EGFR may activate PI3K/AKT pathway and induces resistance, cell proliferation and hypoxia [73]. Dual inhibition of c-MET and EGFR has been shown to efficiently inhibit AKT and MAPK signaling [11,66]. The observed radiosensitizing effect of Pha665752 is mediated through prevention of BRCA1-RAD51 complex formation involved in homologous recombination-mediated DNA repair and persisting unrepaired DNA double strand breaks after irradiation [74]. Clinical use of c-MET targeting in HNSCC was reported so far only within a case study by Chu et al. describing the novel *MET* mutation R1004G as prognostic factor for the response to crizotinib treatment [75].

## 5. Conclusions

The key role of the receptor tyrosine kinase c-MET for cell survival and cancer progression in patients with HNSCC support c-MET as promising target for anticancer therapy. Within the present study we showed that c-MET plays a role for cellular plasticity and stemness in HNSCC, which are major determinants of tumor radioresistance, and provide support for the potential of c-MET targeting strategies for HNSCC radiosensitization. Mechanistic investigation of resistant HNSCC cells to c-MET targeting identified compensatory kinase pathways such as PI3K/AKT and RAS that overcome c-MET blockade and may prevent clinical efficiency in future application. Additional studies are needed to investigate the observed differences between the prognostic potential of *MET* gene expression and phosphorylation status. Overall, these results highlight the importance of comprehensive genomic profiling and biomarker-driven patient stratification to assess and predict the therapeutic potential of targeted therapies in the field of radiooncology.

## Data Availability

Publicly available datasets were analyzed in this study. The used datasets can be found within UCSC Xena (xenabrowser.net) and as GEO accession GSE117973 (Accession viewer, nih.gov).

## Supplementary Materials

The following are available online at https://www.mdpi.com/article/10.3390/cancers13081865/s1, Figure S1: Identification of increased c-MET signaling in radioresistant head and neck squamous cell carcinoma (HNSCC) cell lines and patient data sets; Figure S2: c-MET contributes to cell-intrinsic radiosensitivity in HNSCC cell lines; Figure S3: Cellular plasticity and self-renewal properties of the c-MET-population in HNSCC; Figure S4: c-MET-specific chemical targeting to induce intracellular sensitization of HNSCC cell lines to ionizing radiation.
