# Supplementary material for: Tyrosine Kinase c-MET as Therapeutic Target for Radiosensitization of Head and Neck Squamous Cell Carcinomas
Source: Cancers (Basel). 2021 Apr 14;13(8):1865. doi: 10.3390/cancers13081865 (PMC8070694; doi:10.3390/cancers13081865)

Original Images for Blots/Gels (Lüttich et al.)

Figure 3.

Raw data file: Stephan Heiden – my pictures – WB pics: SAS UT14 XF354 UT45

B

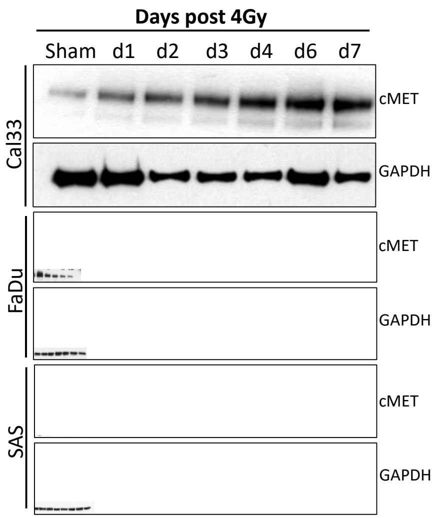

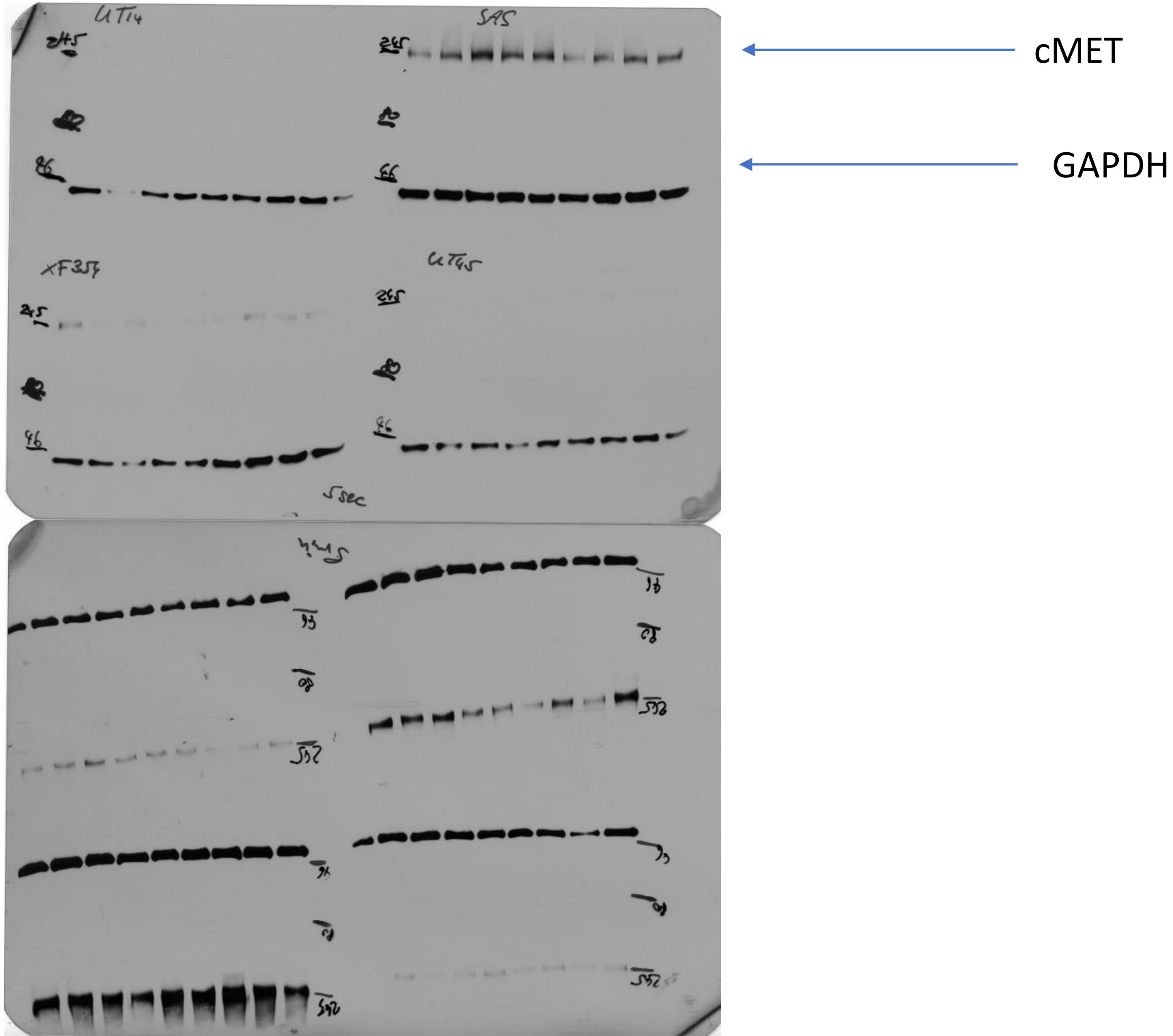

Raw data file: Stephan Heiden – my pictures – WB pics: FaDuRSRR 0-7 1x4Gy

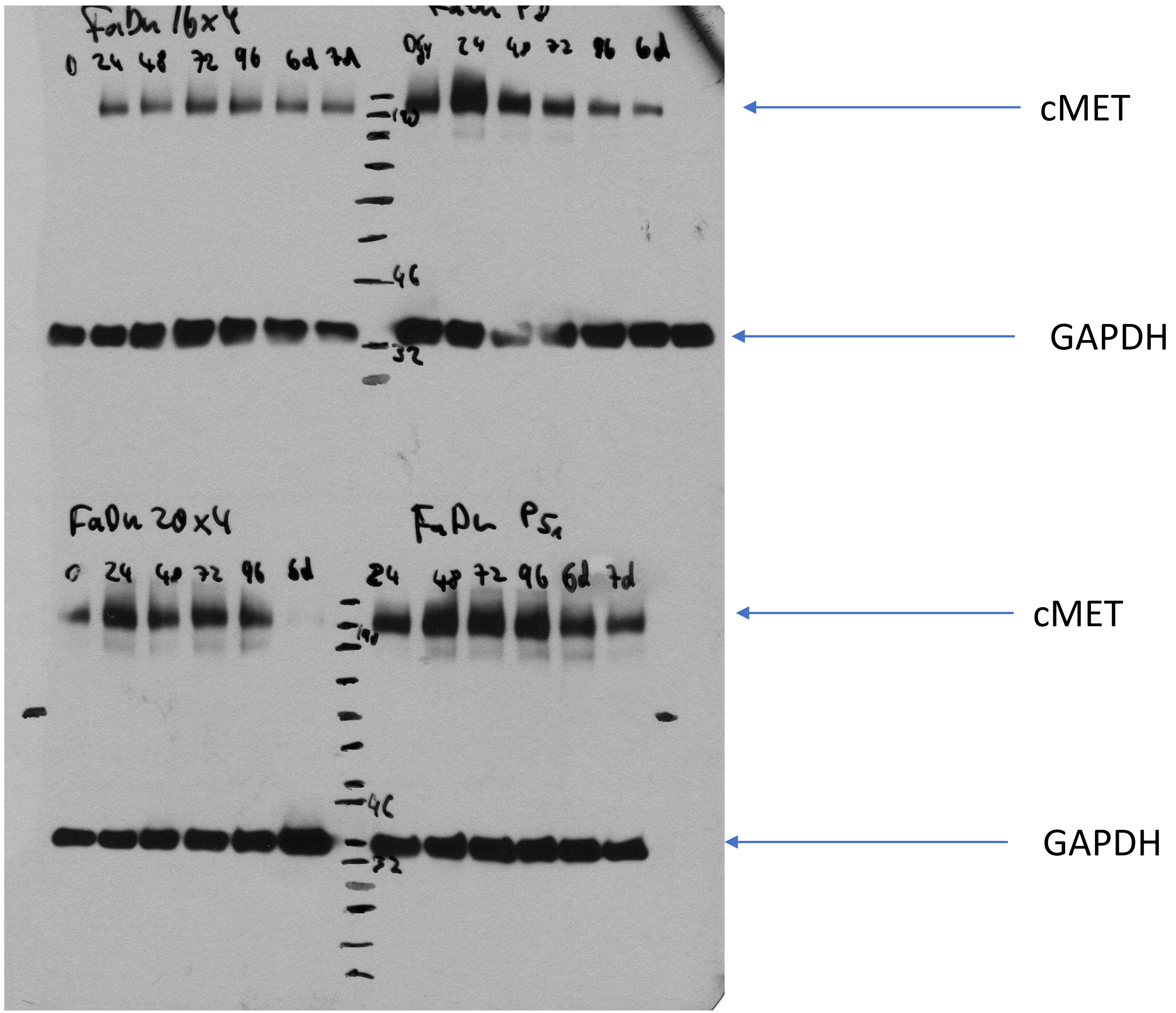

Raw data file: Stephan Heiden – my pictures – WB pics: Cal33 RSRR c-met 0-7 1x4Gy 30 sec

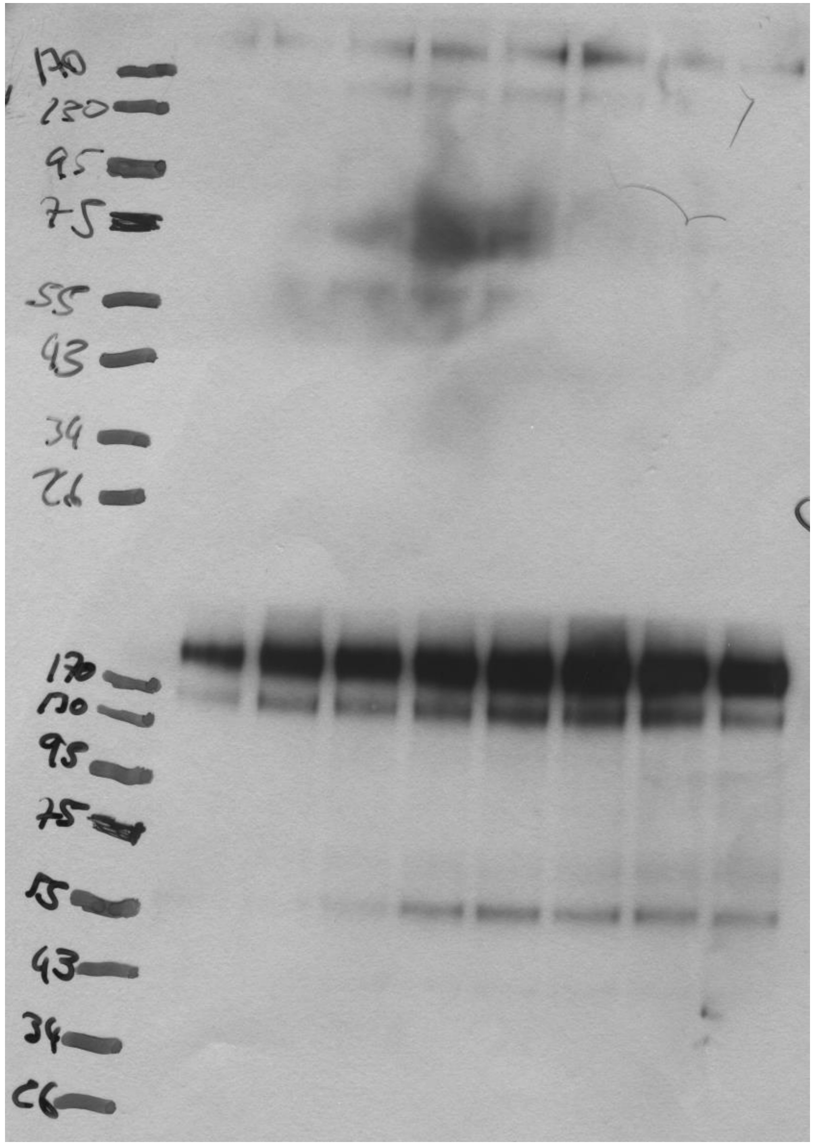

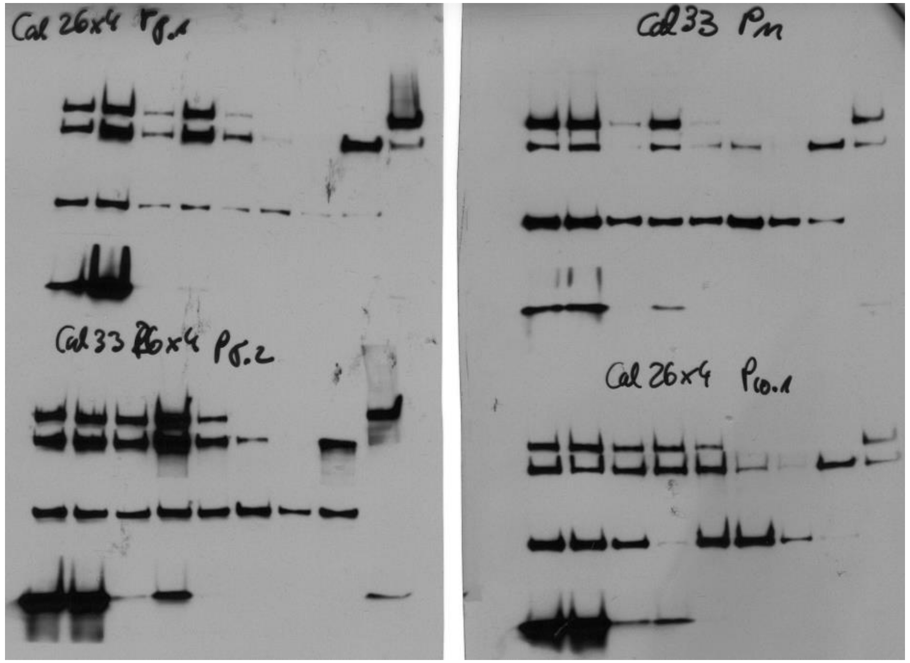

GAPDH

Raw data file: Stephan Heiden – my pictures – WB pics: 30 sec 1x4

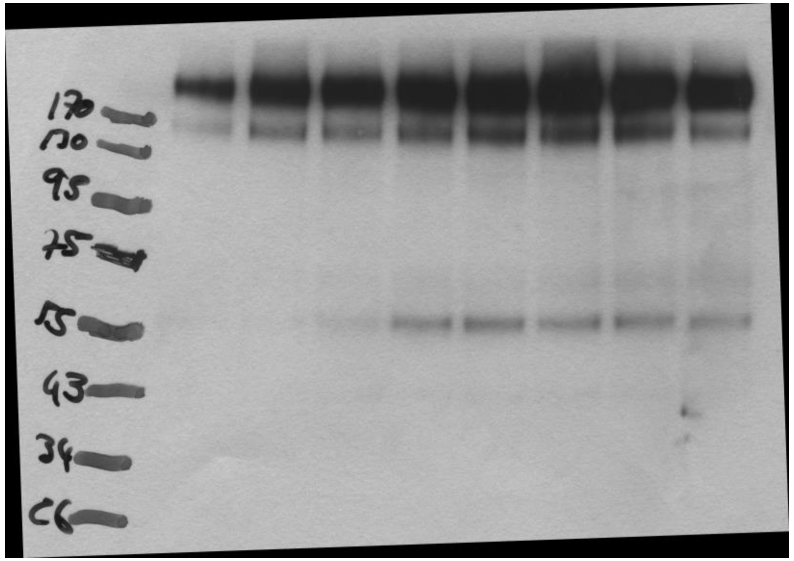

cMET

Figure S3.

B

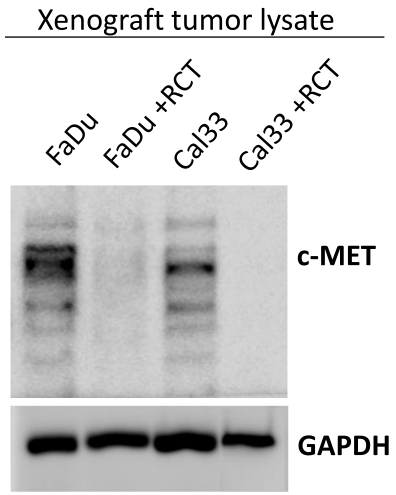

Xenograft tumor lysate
FaDu
FaDu +RCT
Cal33
Cal33 +RCT
c-MET
GAPDH

Previous WB

1st bis-tris gel

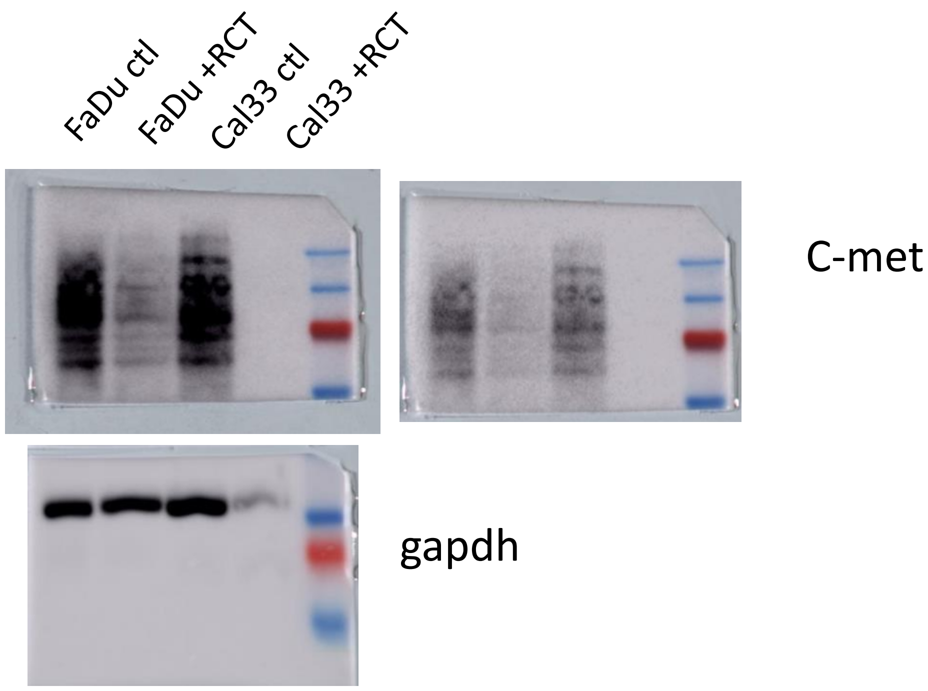

2nd bis-tris gel

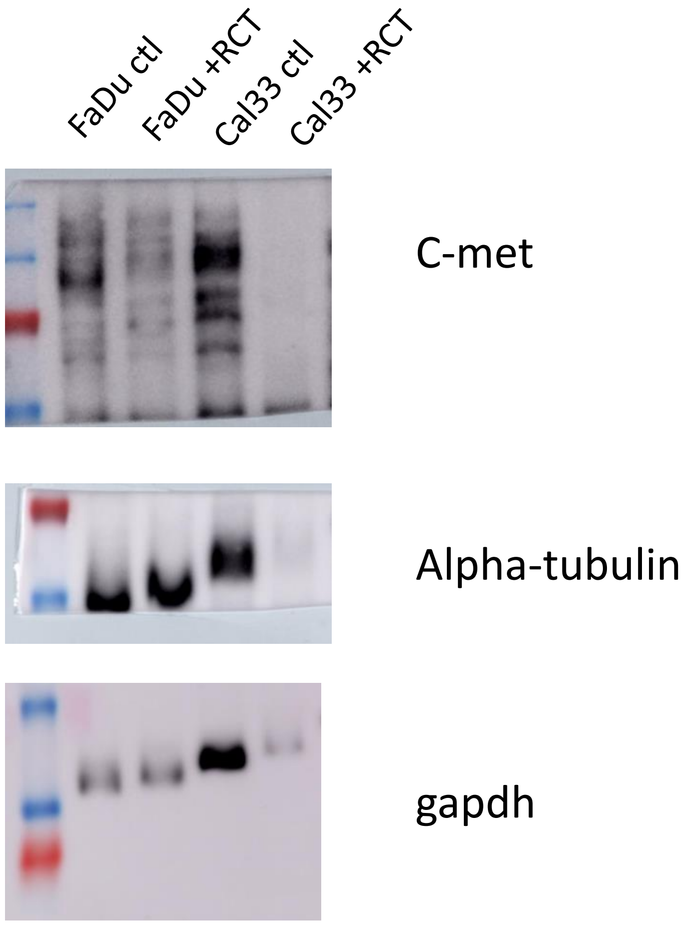

Raw data file: HomeOffice Corona_Collaboration_Ina Maria_WB and IF cmet

bis-tris gel

C-met

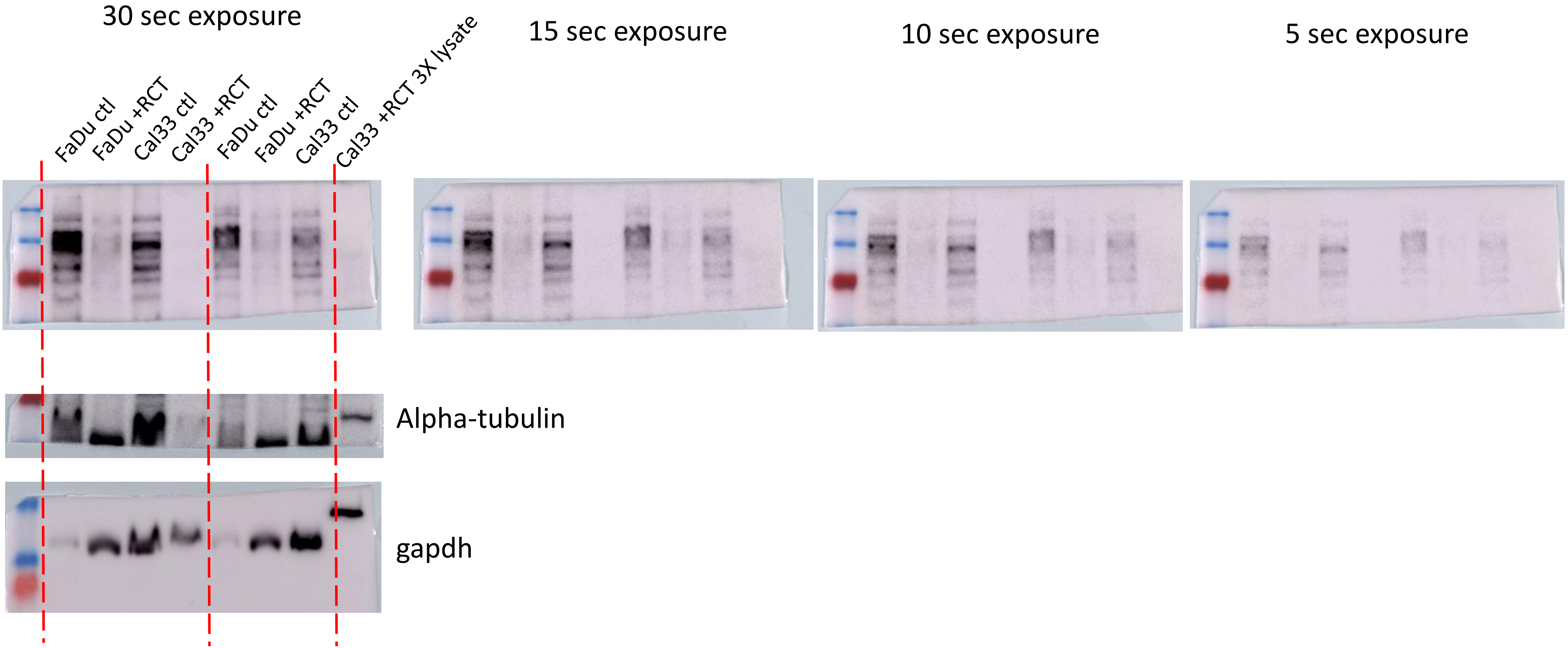

Figure 4.

A

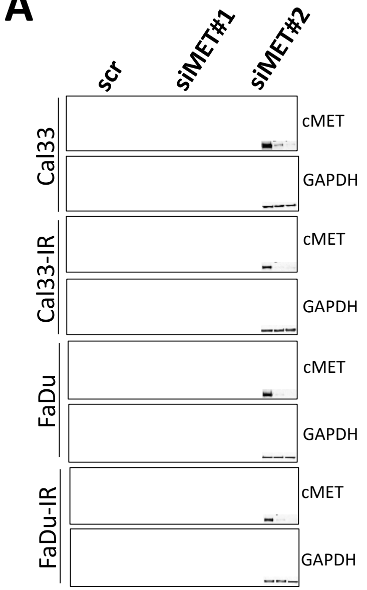

Raw data file: Stephan Heiden – my pictures – WB pics - knockdown colony 1

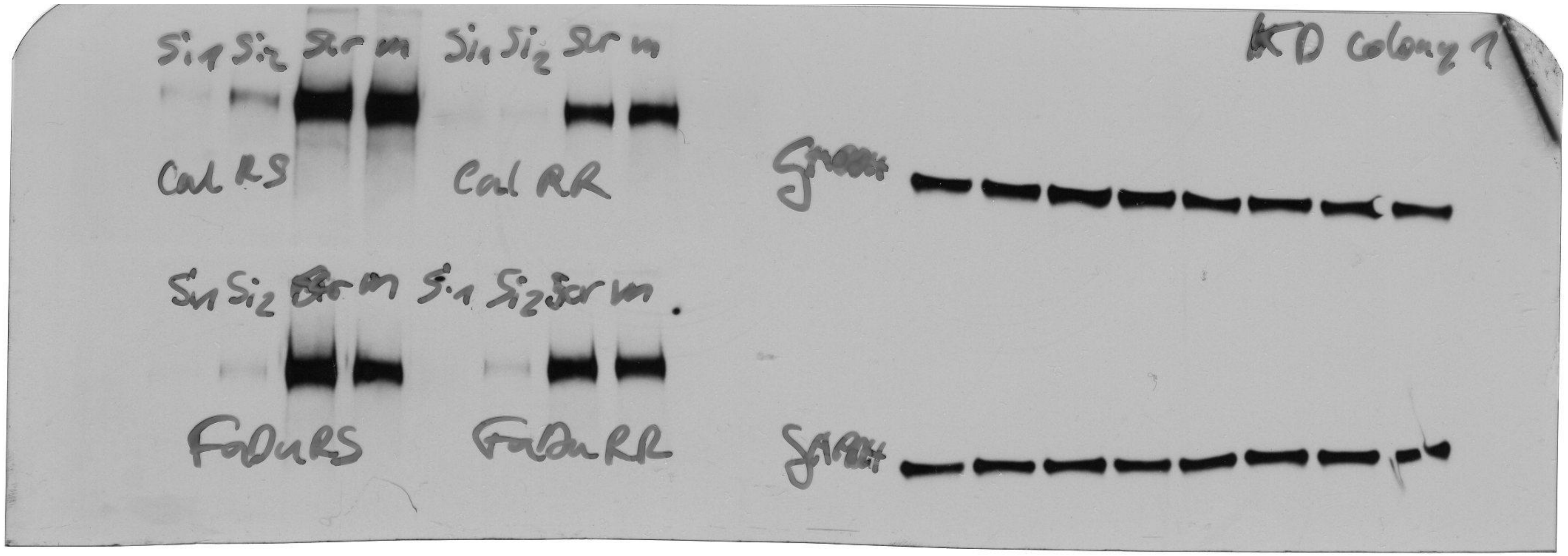

Raw data file: Stephan Heiden – my pictures – WB pics - knockdown colony 2

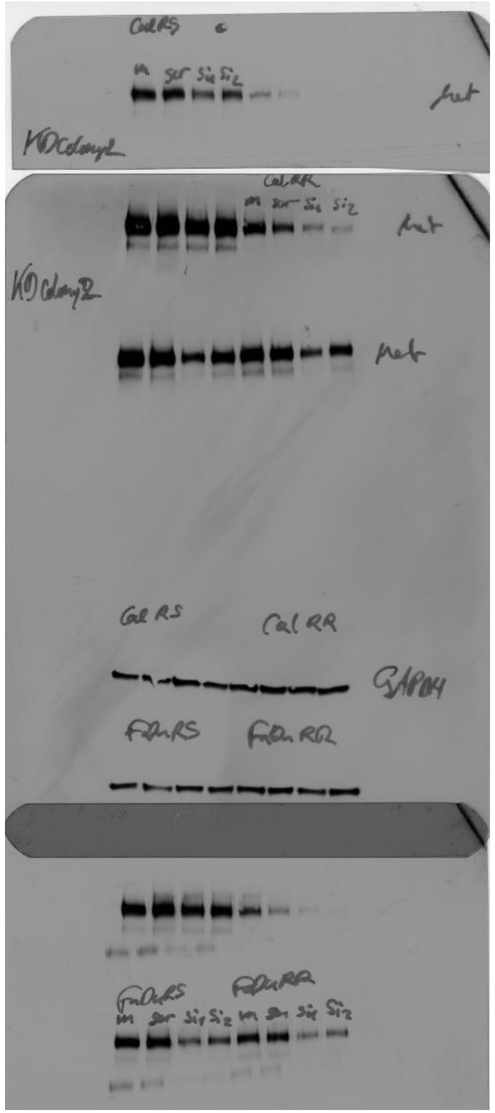

Raw data file: Stephan Heiden – my pictures – WB pics - knockdown colony 3

cMET

GAPDH

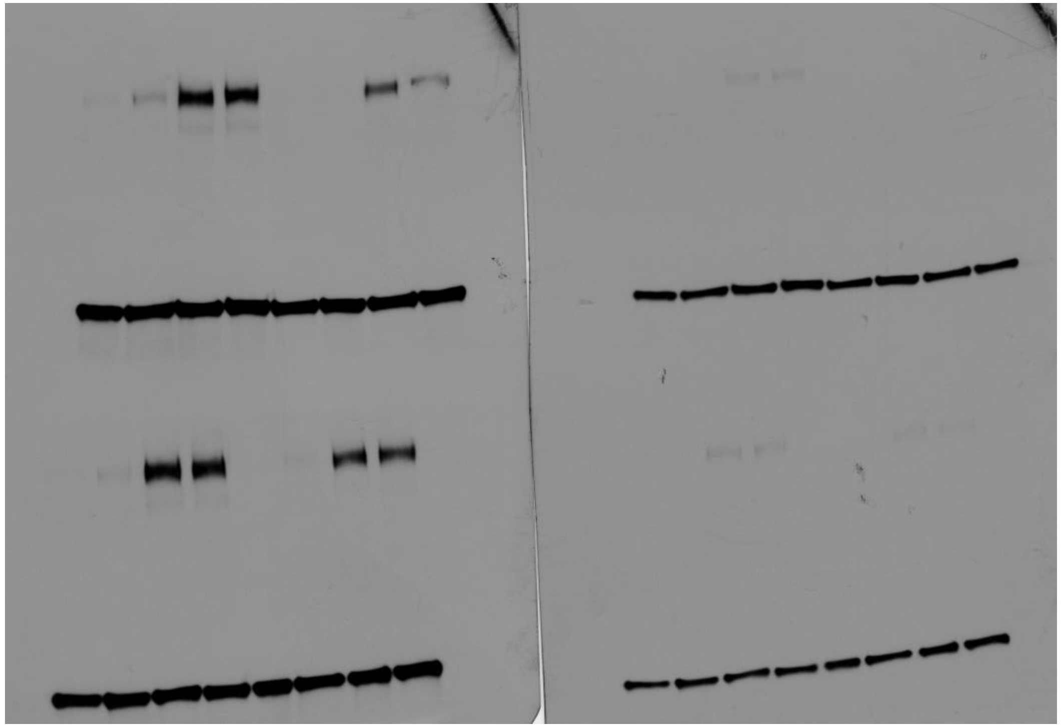

Figure 4.

E

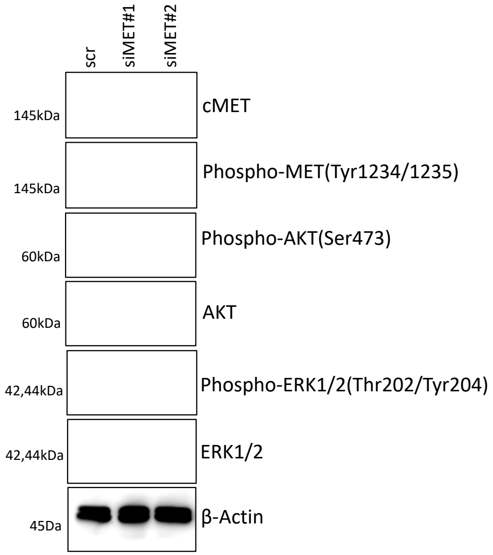
scr
siMET#1
siMET#2
145kDa
cMET
145kDa
Phospho-MET(Tyr1234/1235)
60kDa
Phospho-AKT(Ser473)
60kDa
AKT
42,44kDa
Phospho-ERK1/2(Thr202/Tyr204)
42,44kDa
ERK1/2
45Da
β-Actin

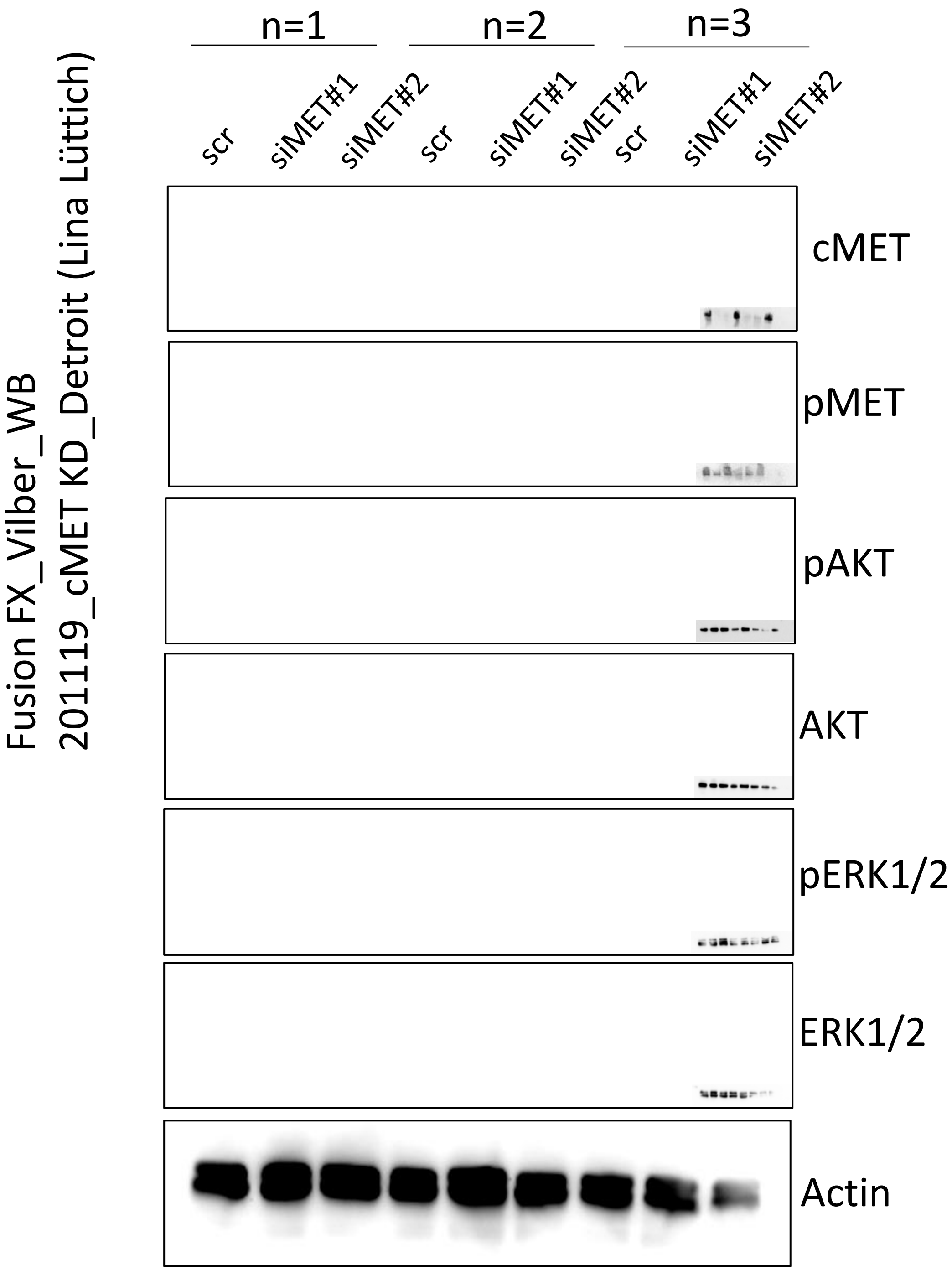
Fusion FX_Vilber_WB
201119_cMET KD_Detroit (Lina Lüttich)
n=1
n=2
n=3
scr
siMET#1
siMET#2
scr
siMET#1
siMET#2
scr
siMET#1
siMET#2
cMET
pMET
pAKT
AKT
pERK1/2
ERK1/2
Actin

201119_cMET

MET_04Sum

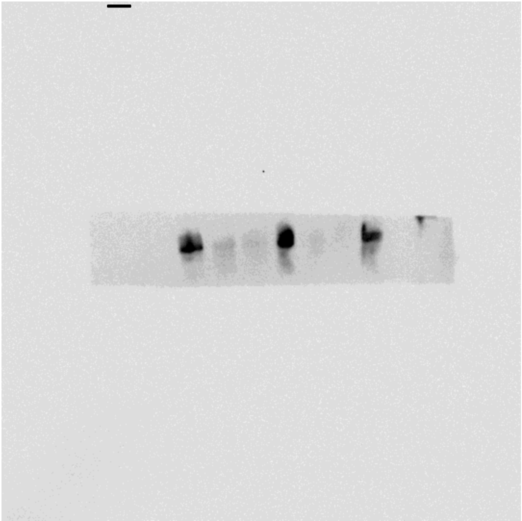

Actin_8

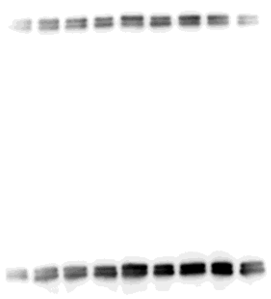

pMET_05Sum

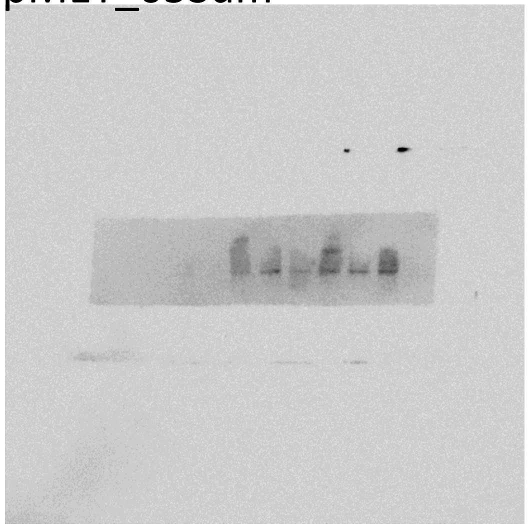

pAKT_04Sum

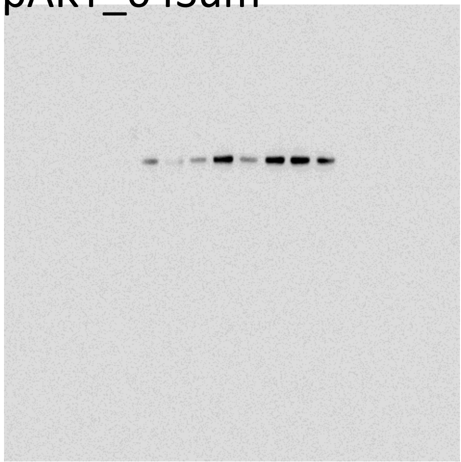

Marker_2B

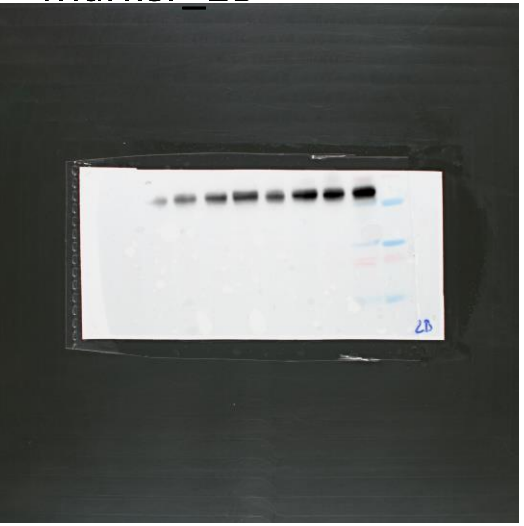

AKT_05Sum

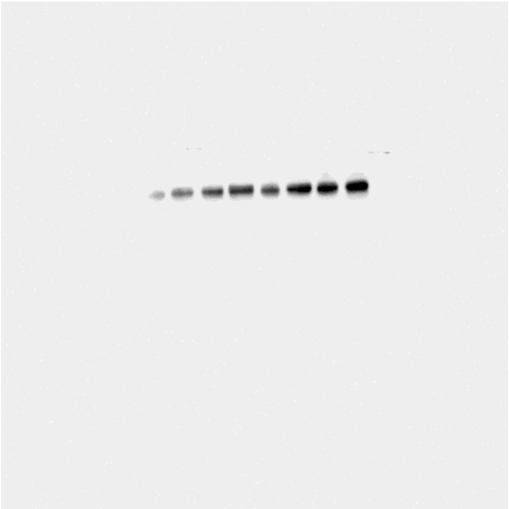

pERK_05Sum

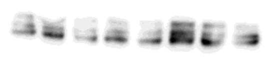

ERK_03

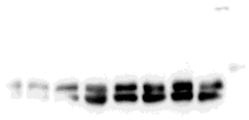

Figure 5.

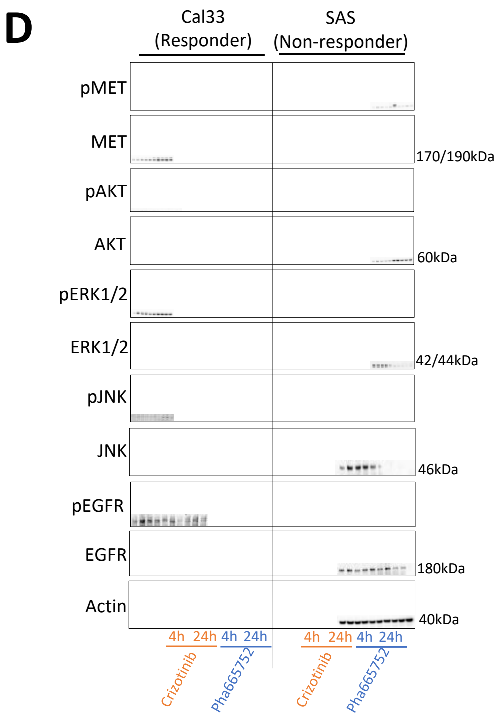

Raw data: 10_April19_Febr20 – Data - WB

cMET_5(Chemiluminescence)

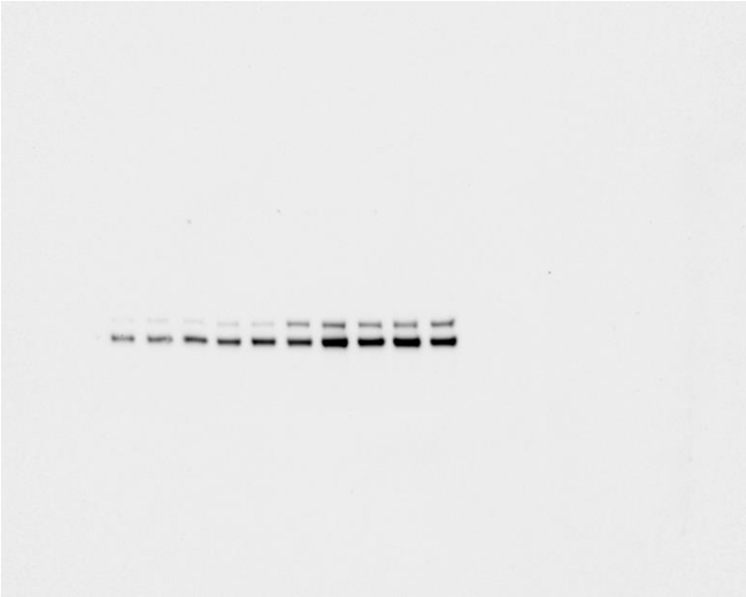

AKT_6(Chemiluminescence)

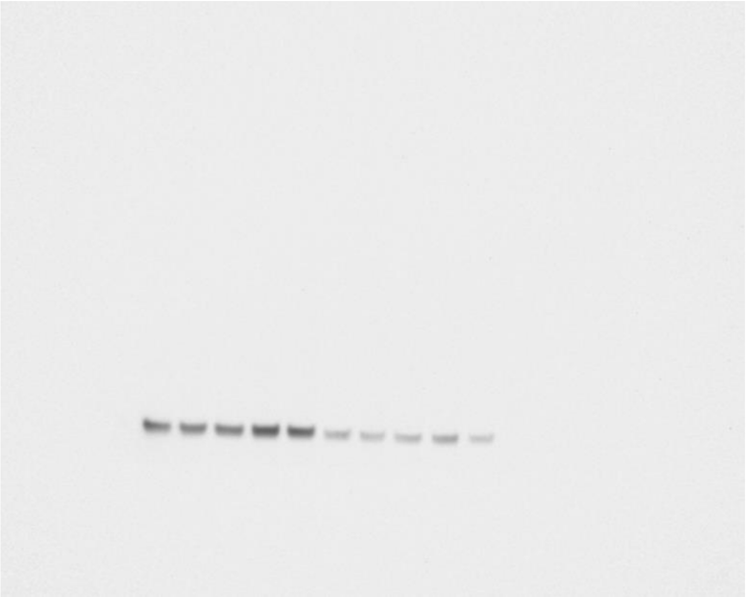

cMET_5(Composite)

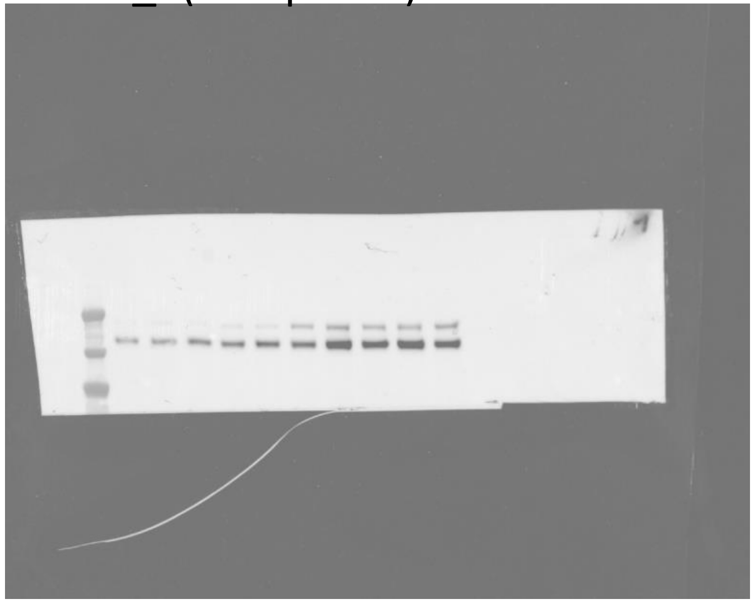

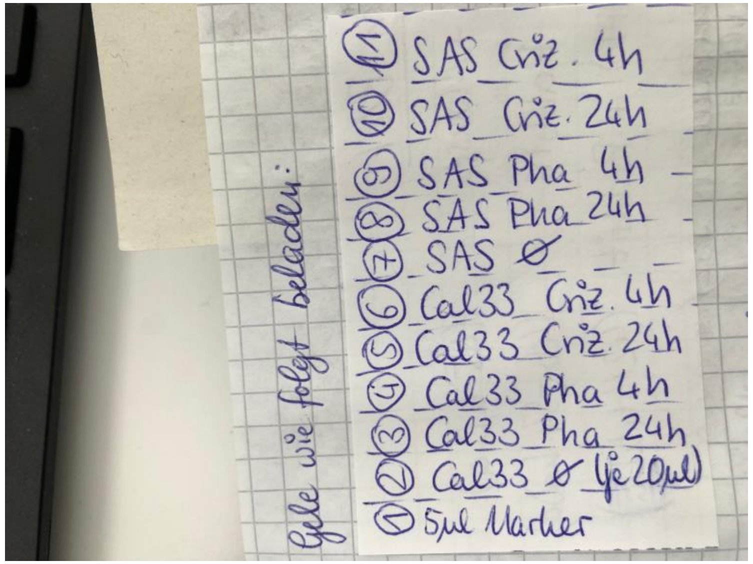

pEGFR Y11735_8(Chemiluminescence)

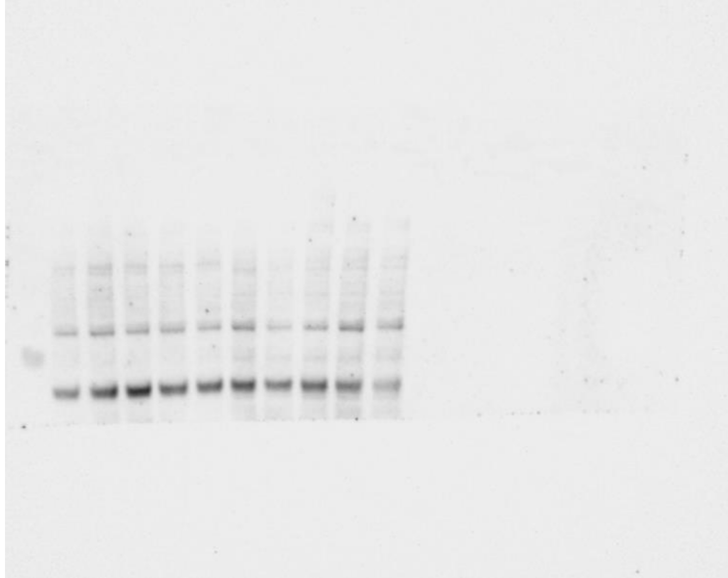

AKTIN_6(Chemiluminescence)

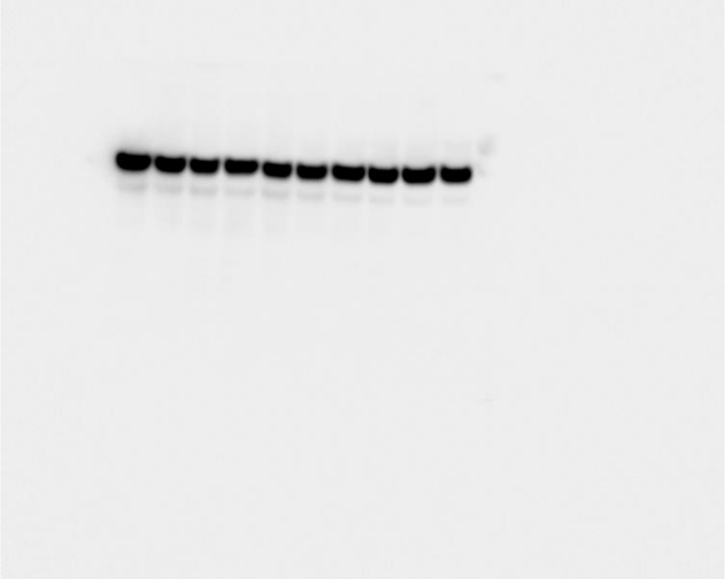

EGFR ab_5(Chemiluminescence)

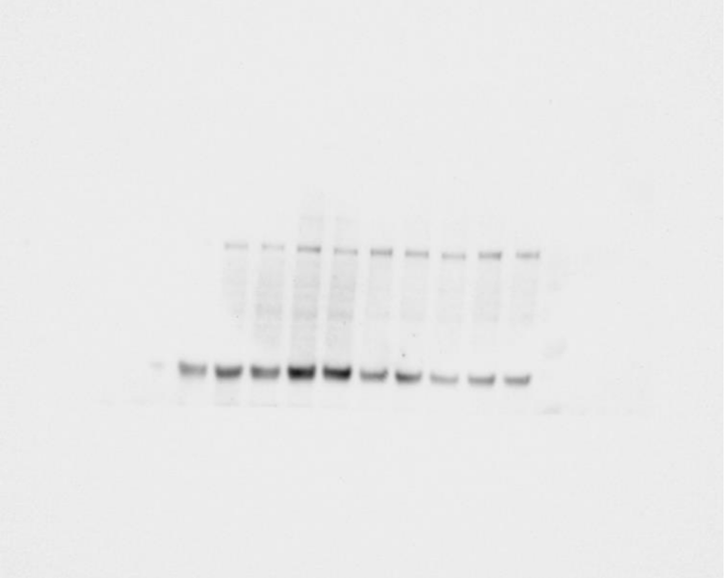

ERK_10(Chemiluminescence)

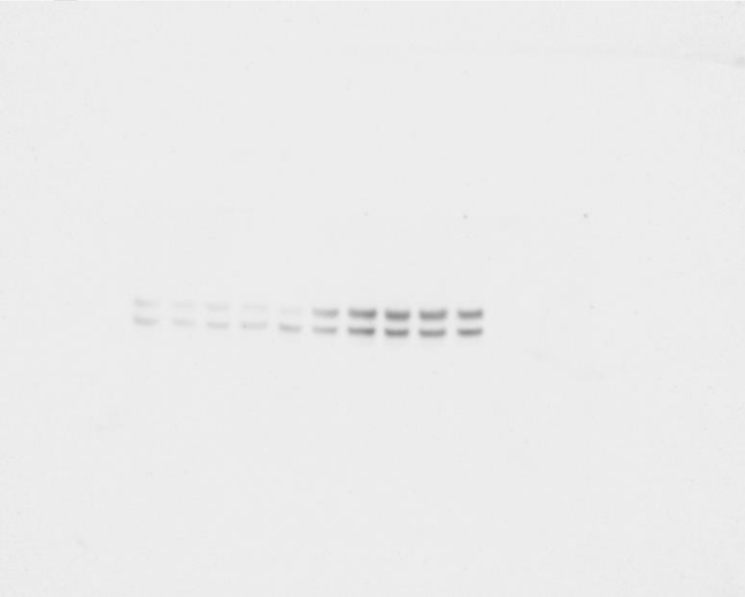

ERK_11(Colorimetric)

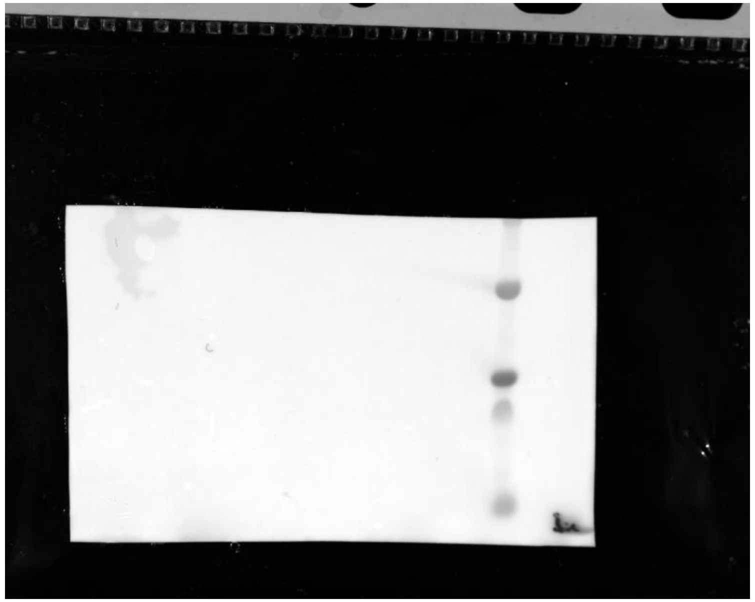

pMET_6(Chemiluminescence)

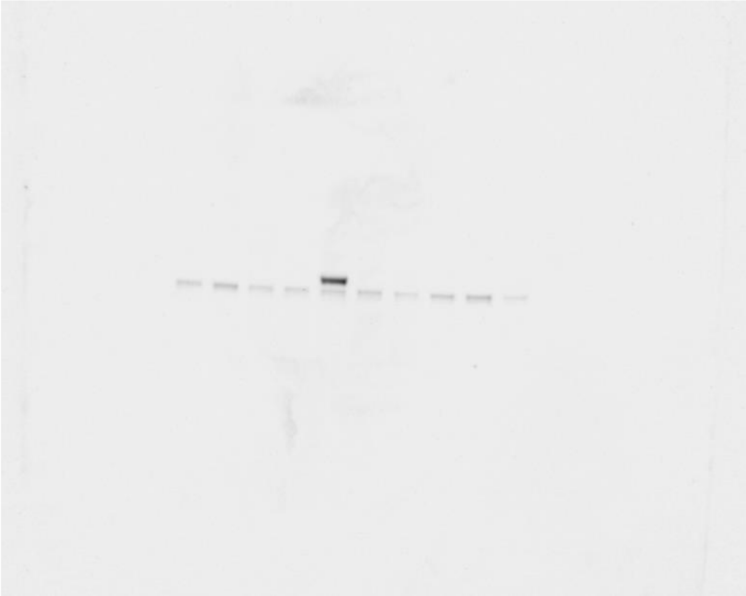

Supplement: Supplementary file 1 [file cancers-13-01865-s001.zip › cancers-1144378-supplmentary/cancers-1144378-original images.pdf]
