# Supplementary material for: Tyrosine Kinase c-MET as Therapeutic Target for Radiosensitization of Head and Neck Squamous Cell Carcinomas
Source: Cancers (Basel). 2021 Apr 14;13(8):1865. doi: 10.3390/cancers13081865 (PMC8070694; doi:10.3390/cancers13081865)
Supplement: Supplementary file 1 [file cancers-13-01865-s001.zip › cancers-1144378-supplmentary/cancers-1144378-suppl-final.docx]

Supplemental Materials: Tyrosine Kinase c-MET as Therapeutic Target for Radiosensitization of Head and Neck Squamous Cell Carcinomas

Lina Lüttich, María José Besso, Stephan Heiden, Lydia Koi, Michael Baumann, Mechthild Krause, Anna Dubrovska, Annett Linge, Ina Kurth and Claudia Peitzsch


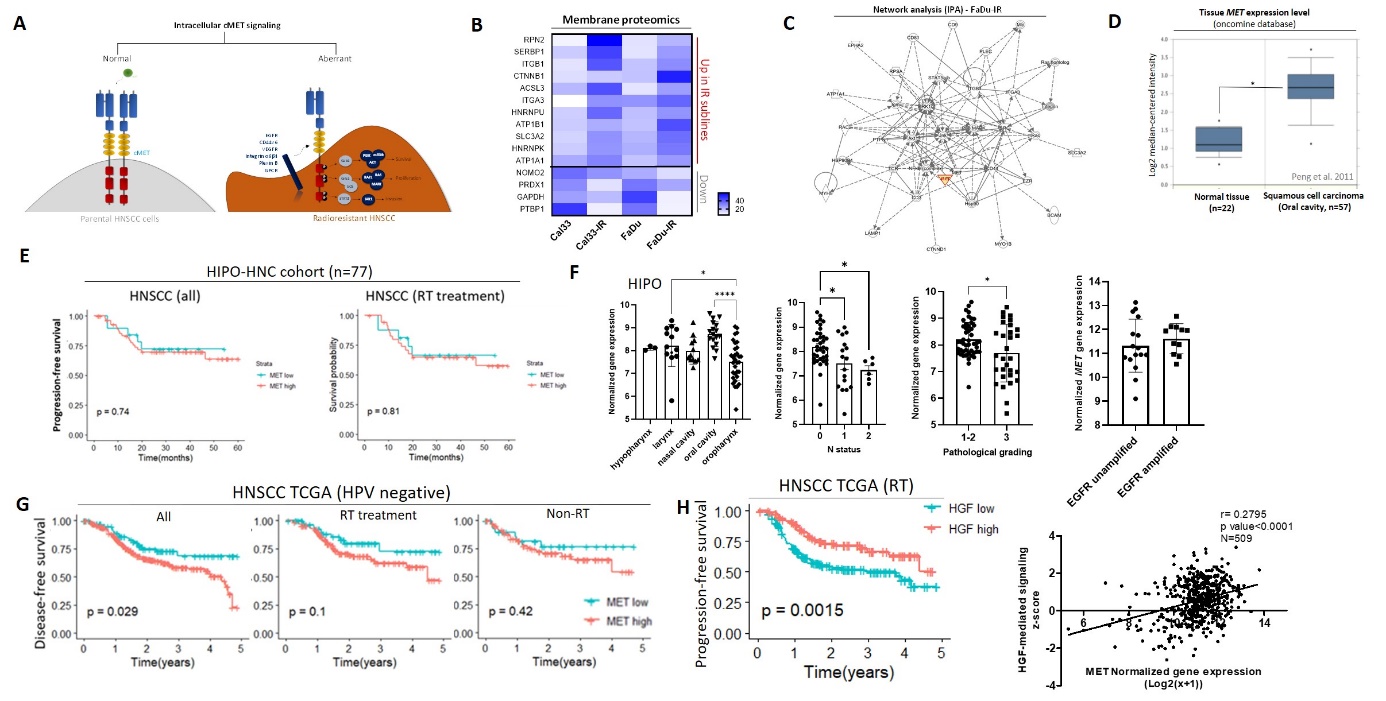


**Figure S1.** Identification of increased c-MET signaling in radioresistant head and neck squamous cell carcinoma (HNSCC) cell lines and patient data sets. (**A**) Schematic illustration of altered intracellular c-MET signaling in the generated HNSCC-IR sublines that involves Gab1-PI3K7AKT/mTOR signaling induced cell survival, Grb/SOS-RAF1/RAS/MAPK regulated cell proliferation and STAT3/JAK1 mediated cell invasion. Moreover, interaction with other receptor tyrosine kinase receptors such as EGFR and VEGFR or other cell membrane proteins including CD44 and integrin-mediating activation of intracellular c-MET signaling in radioresistant HNSCC cells is shown. (**B**) Proteomic analysis comparing IR-sublines to parental control in Cal33 and FaDu cells identified 11 up- and 4 down-regulated proteins (*n* = 3, *p* < 0.05, fold change >1.5). (**C**) Differential regulated genes in FaDu-IR subline compared to control were functionally connected using network analysis tool of Ingenuity pathway analysis (IPA) software. (**D**) Oncomine database analysis illustrates higher MET expression in squamous cell carcinoma that originate from the oral cavity (*n* = 57) compared to adjacent normal tissue (*n* = 22). (**E**) To validate the results from TCGA study we analyzed in silico the publicly available data from Heidelberg Center for Personalized Oncology – head and neck cancer cohort (HIPO-HNC, *n* = 77). Within this study MET expression does not have a prognostic potential independent of the treatment. The lack of concordance and significancy is influenced by the small cohort size and does not allow for subgroup analysis**. (F)** *MET* expression in HIPO-HNC study was significantly correlated to tumor localization, nodal (N) status and pathological grading while no association with *EGFR* was seen. (G) Subgroup analysis of TCGA dataset for patients with HPV-negative HNSCC validated the prognostic potential of *MET*. **(H)** Kaplan-Meier curves illustrates progression-free survival analysis of patients with HNSCC treated with radiotherapy (RT) from the TCGA cohort that were stratified based the hepatocyte growth factor (*HGF*) transcript level (** *p* = 0.00015). There is a positive correlation between *MET* transcript levels and HGF signaling (*n* = 505, r = 0.279, *** *p* < 0.001).


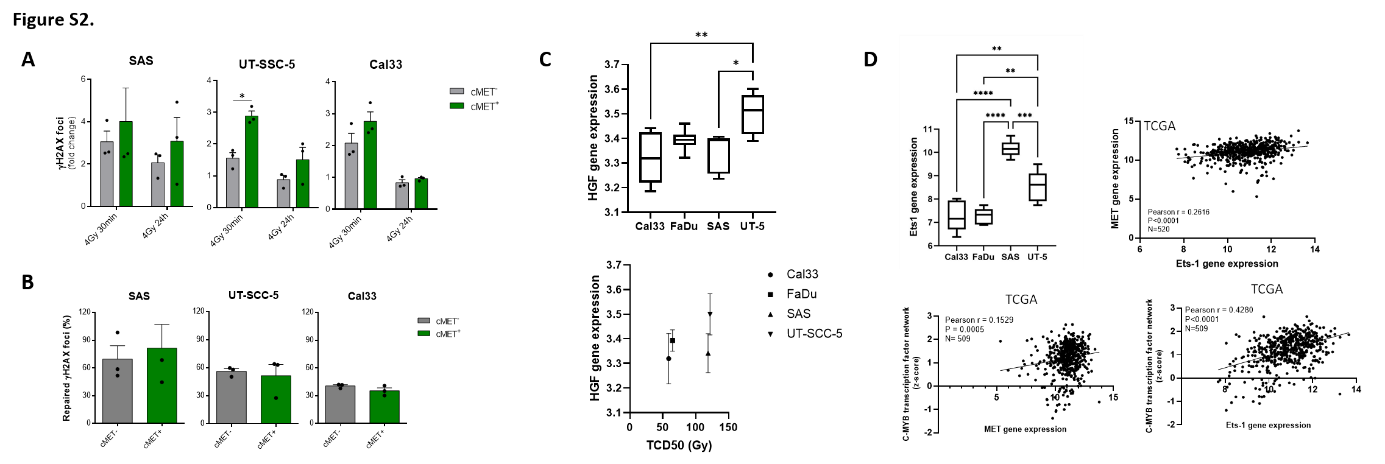


**Figure S2.** c-MET contributes to cell-intrinsic radiosensitivity in HNSCC cell lines. (**A,B**) c-MET high and low expressing populations from HNSCC cell lines SAS, UT-SCC-5 and Cal33 were purified using fluorescence-activated cell sorting (FACS) and plated for yH2AX foci assay. Therefore, cells were irradiated with 4 Gy upon adhesion to microscopic glass slides and fixed after 30 min and 24 h to determine initial and residual foci indicative of DNA double strand breaks. To illustrate differences in DNA repair capacity between the c-MET high and low population, determined yH2AX foci per cell were normalized to baseline counts (a) and to initial damages 30 min after irradiation (b) (*n* = 3, * *p* < 0.05, mean ± SEM). (**C**) Gene expression analysis of *HGF* in Cal33, FaDu, SAS and UT-SCC-5 derived xenograft tumors that were further correlated to previously published tumor control dose 50% (TCD50) values. (**D**) Transcriptomic analysis of *ETS1* and c-Myb in HNSCC xenograft tumors and within the TCGA dataset from HNSCC patients.


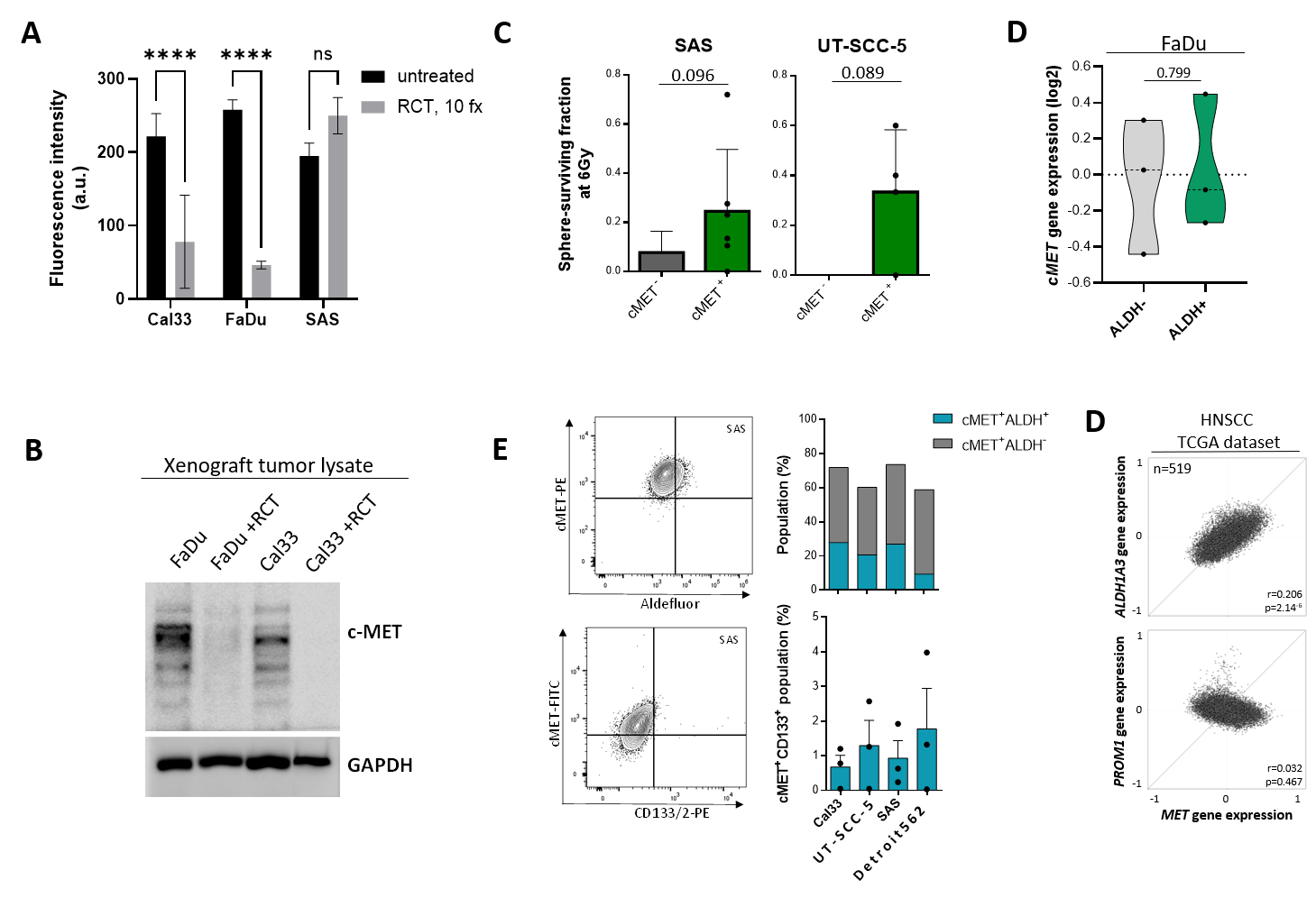


**Figure S3.** Cellular plasticity and self-renewal properties of the c-MET-population in HNSCC. (**A**) Quantification of c-MET signal within tissue sections of Cal33, FaDu and SAS xenograft tumor that were treated with radio(chemo)therapy (RCT) in comparison to untreated tumors through automated determination of mean pixel intensity (arbitrary unit (a.u.), *n* = 3, two-way ANOVA, **** *p* < 0.00001). (**B**) Western blot analysis to determine c-MET expression in xenograft tumor lysates. GAPDH was used as loading control (*n* = 2). (**C**) Prospectively purified c-MET-high and low expressing population from SAS and UT-SCC-5 cells for sphere formation. The representative Celigo images illustrate all formed spheres from initially plated 1000 cells. To evaluate cell intrinsic radiosensitivity of spheres originating from MET^high^ and MET^low^ population the sphere-forming capacity upon 6 Gy irradiation was normalized to sham control (*n* = 3). (**D**) *MET* gene expression analysis purified Aldefluor (ALDH)-positive FaDu cells compared to the ALDH-negative population based on Agilent array data (*n* = 3). (**E**) Co-expression of c-MET with the CSC markers CD133 and Aldefluor (ALDH) using multicolor flow cytometry (BD FACS Celesta) in the HNSCC cell lines Cal33, SAS, UT-SCC-5 and Detroit562 (*n* = 3). (**F**) In silico correlation analysis of TCGA dataset for HNSCC patients (*n* = 517) demonstrated no association of *MET* gene expression with the CSC markers *ALDH1A3* and *PROM1* (CD133 gene) (Pearson correlation).


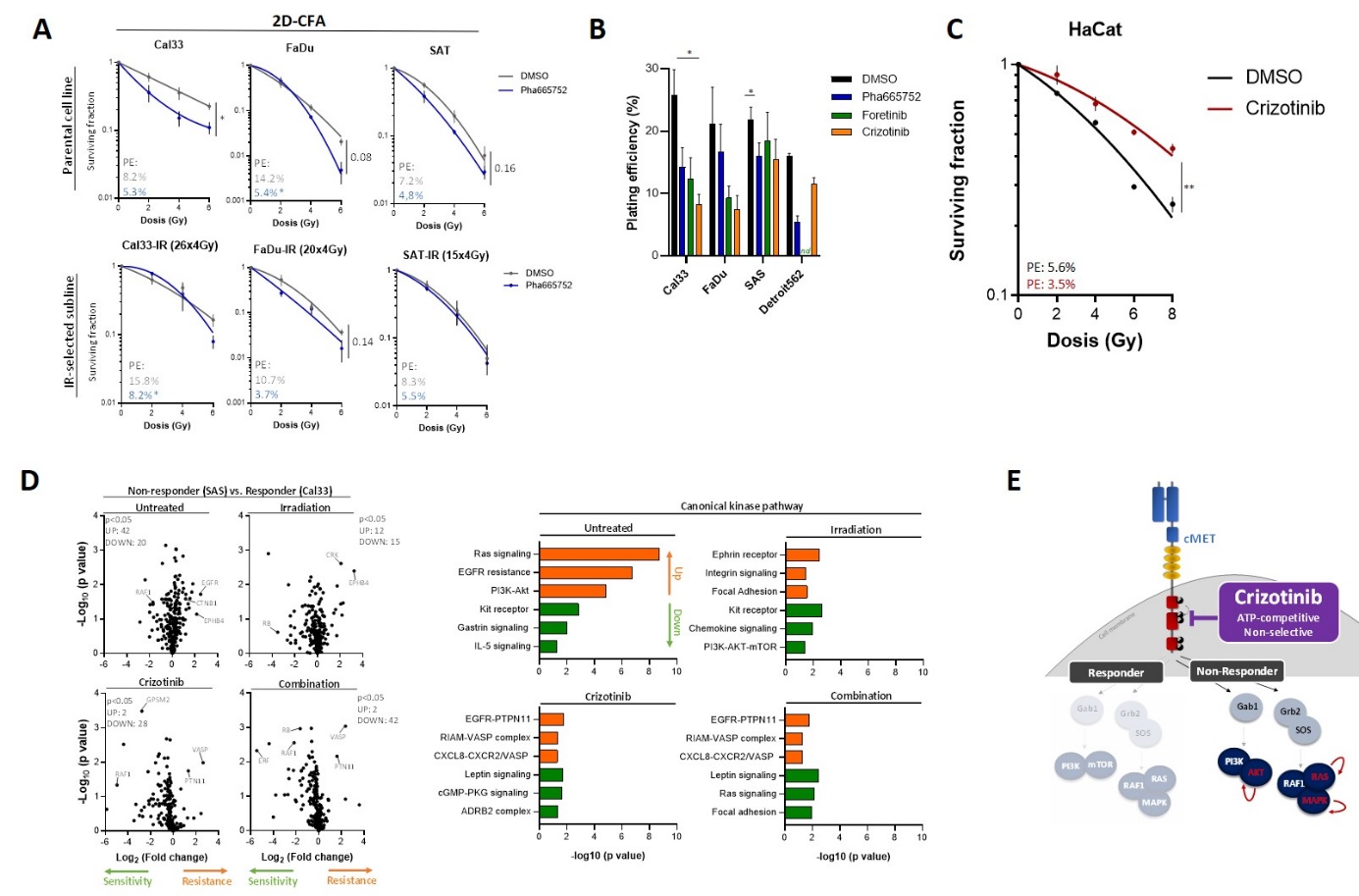


**Figure S4.** c-MET-specific chemical targeting to induce intracellular sensitization of HNSCC cell lines to ionizing radiation. (**A**) The HNSCC cell lines Cal33, FaDu and SAS as well as their irradiated sublines were pre-treated for 1 h with IC50 concentration (1.5 µM) of the c-MET inhibitor Pha665752 in comparison to DMSO vehicle control, plated for 2D-colony formation assay and irradiated with increasing doses from 2–6 Gy. The dose-response curves illustrate a decreased clonogenic survival and in the parental cell lines a moderate radiosensitization through Pha665752 (*n* = 3, mean ± SEM, * *p* < 0.05). (**B**) Influence of c-MET inhibitors Pha665752, foretinib and crizotinib on clonogenic survival in 3D-matrigel-based colony formation assay for Cal33 and SAS cells (*n* = 3, mean ± SEM, * *p*<0.05). (**C**) Crizotinib is decreasing the radiosensitivity of the keratinocyte cell line HaCaT significantly (*n* = 3, mean ± SEM, **p < 0.01). (**D**) Comparative kinome activity analysis of responding cell line Cal33 in comparison to non-responding line SAS, including upstream kinase signaling pathway analysis. (**E**) Representative scheme illustrating the observed resistance to crizotinib treatment in HNSCC cells mediated by compensatory PI3K/AKT and RAS/RAF/MAPK signaling.
